# Distinct Clones of *Yersinia pestis* Caused the Black Death

**DOI:** 10.1371/journal.ppat.1001134

**Published:** 2010-10-07

**Authors:** Stephanie Haensch, Raffaella Bianucci, Michel Signoli, Minoarisoa Rajerison, Michael Schultz, Sacha Kacki, Marco Vermunt, Darlene A. Weston, Derek Hurst, Mark Achtman, Elisabeth Carniel, Barbara Bramanti

**Affiliations:** 1 Institute for Anthropology, Johannes Gutenberg University, Mainz, Germany; 2 Laboratory of Criminalistic Sciences Department of Anatomy, Pharmacology and Legal Medicine, University of Turin, Turin, Italy; 3 Unité d'Anthropologie Bioculturelle, Faculté de Medecine, University of Mediterranean-CNRS-EFS, Marseille, France; 4 Centre d'Études Préhistoire, Antiquité, Moyen-âge, UMR 6130 CNRS–250 University of Nice, Valbonne, France; 5 Center for Plague, Institute Pasteur de Madagascar, World Health Organization Collaborating, Antananarivo, Madagascar; 6 Department of Anatomy and Embryology Medical Faculty, Georg-August University, Göttingen, Germany; 7 Inrap, Villeneuve-d'Ascq Archaeological Center, Villeneuve-d'Ascq, France; 8 Laboratoire d'Anthropologie des Populations du Passé, Université Bordeaux 1, Talence, France; 9 Department of Monuments and Archaeology, Municipality of Bergen op Zoom, Bergen op Zoom, The Netherlands; 10 Barge's Anthropologica, Department of Anatomy and Embryology, Leiden University Medical Center, Leiden, The Netherlands; 11 Division of Archaeological Sciences, University of Bradford, Bradford, West Yorkshire, United Kingdom; 12 Department of Human Evolution, Max Planck Institute for Evolutionary Anthropology, Leipzig, Germany; 13 Worcestershire Historic Environment and Archaeology Service, Worcestershire County Council, Worcester, United Kingdom; 14 Environmental Research Institute, University College Cork, Cork, Ireland; 15 Yersinia Research Unit, Institut Pasteur, Paris, France; University of Notre Dame, United States of America

## Abstract

From AD 1347 to AD 1353, the Black Death killed tens of millions of people in Europe, leaving misery and devastation in its wake, with successive epidemics ravaging the continent until the 18^th^ century. The etiology of this disease has remained highly controversial, ranging from claims based on genetics and the historical descriptions of symptoms that it was caused by *Yersinia pestis* to conclusions that it must have been caused by other pathogens. It has also been disputed whether plague had the same etiology in northern and southern Europe. Here we identified DNA and protein signatures specific for *Y. pestis* in human skeletons from mass graves in northern, central and southern Europe that were associated archaeologically with the Black Death and subsequent resurgences. We confirm that *Y. pestis* caused the Black Death and later epidemics on the entire European continent over the course of four centuries. Furthermore, on the basis of 17 single nucleotide polymorphisms plus the absence of a deletion in *glpD* gene, our aDNA results identified two previously unknown but related clades of *Y. pestis* associated with distinct medieval mass graves. These findings suggest that plague was imported to Europe on two or more occasions, each following a distinct route. These two clades are ancestral to modern isolates of *Y. pestis* biovars Orientalis and Medievalis. Our results clarify the etiology of the Black Death and provide a paradigm for a detailed historical reconstruction of the infection routes followed by this disease.

## Author Summary

Several historical epidemic waves of plague have been attributed to *Yersinia pestis*, the etiologic agent of modern plague. The most famous of these was the second pandemic which was active in Europe from AD 1347 until 1750, and began with the ‘Black Death’. The most informative method to establish the etiological nature of these ancient infections should be the analysis of ancient DNA, but the results of this method have been controversial. Here, by combining ancient DNA analyses and protein-specific detection, we demonstrate unambiguously that *Y. pestis* caused the Black Death. Furthermore, we show that at least two variants of *Y. pestis* spread over Europe during the second pandemic. The analysis of up to 20 diagnostic markers reveals that the two variants evolved near the time that phylogenetic branches 1 and 2 separated and may no longer exist. Our results thus resolve a long-standing debate about the etiology of the Black Death and provide key information about the evolution of the plague bacillus and the spread of the disease during the Middle Ages.

## Introduction

Of the numerous epidemics in human history, three pandemics are generally accepted as having been caused by plague. Justinian's plague (AD 541–542) spread from Egypt to areas surrounding the Mediterranean [1]. In 1347, an epidemic known as the Black Death spread from the Caspian Sea to almost all European countries, causing the death of one third of the European population over the next few years [2]. This second pandemic persisted in Europe until 1750, causing successive and progressively declining epidemic waves. A third plague pandemic began in the Yunnan region of China in the mid-19^th^ century, and spread globally via shipping from Hong Kong in 1894. During this last pandemic, the etiological cause of plague was identified as *Yersinia pestis*, a Gram-negative bacterium [3], [4]. Most microbiologists and epidemiologists believe that *Y. pestis* was also the etiological agent of the first two pandemics. This belief is supported by ancient DNA (aDNA) analyses which identified sequences specific for *Y. pestis* in the teeth of central European plague victims from the first and second pandemics [5]–[7]. Moreover, the *Y. pestis* F1 protein capsule antigen has been detected in ancient plague skeletons from Germany and France by immunochromatography [8], [9].

Based on studies on modern strains, microbiologists have subdivided *Y. pestis* into three biovars: Antiqua, Medievalis, and Orientalis. These biovars can be distinguished depending on their abilities to ferment glycerol and reduce nitrate [10]. The Medievalis biovar is unable to reduce nitrates due to a G to T mutation that results in a stop codon in the *napA* gene [11], while the Orientalis biovar cannot ferment glycerol because of a 93 bp deletion in the *glpD* gene [11], [12]. Conversely, the Antiqua biovar is capable of performing both reactions [10]. An apparent historical association of the routes of the three pandemics with the modern geographical sources of the three biovars led Devignat to propose that each plague pandemic was caused by a different biovar [10]. There is no doubt that the ongoing third pandemic was caused by biovar Orientalis, but an attribution of the first and second pandemics to Antiqua and Medievalis, respectively, is questionable. Unlike Devignat's hypothesis, recent aDNA analyses of samples from the 7^th^–9^th^ and 18^th^ centuries yielded Orientalis-specific microsatellites [13] and the characteristic 93 bp *glpD* deletion [14], thus suggesting that the Orientalis biovar also caused Justinian's plague and the second pandemic.

Despite these results, a debate continues regarding whether *Y. pestis* really was the causative agent of the Black Death, as summarized by Byrne [15]. Some epidemiologists and historians have denied this conclusion due to inconsistencies between the clinical and epidemiological characteristics of plague in historical records and those observed in India in the early 20^th^ century [16]–[19]. Alternative putative etiologies of the Black Death include a viral hemorrhagic fever [16] or a currently unknown pathogen [19]. In part, these alternative etiologies reflect apparent discrepancies between historical observations of extremely rapid spread of mortality during the Black Death with the dogma based on Indian epidemiology that plague is associated with transmission from infected rats via blocked fleas, which can first transmit *Y. pestis* approximately 30-days after a blood meal. However, recent data show that transmission by fleas can occur continuously after a blood meal and does not depend on blockage [20]. Even the aDNA studies have not been considered to be conclusive because other teams were initially unable to find ancient *Y. pestis* DNA in human teeth from plague pits in central and north Europe [21], thus supporting the interpretation that the massive human deaths that occurred in these areas at the time of the second pandemic were not caused by *Y. pestis*.

Here, we combined analyses of aDNA with detection of the *Y. pestis* F1 antigen on skeletons from mass graves throughout Europe that date to the time of the Black Death and later epidemic waves. Our results show that historical plague was caused by *Y. pestis* throughout Europe. We also genotyped the causative agent for these mass fatalities by informative single nucleotide polymorphisms (SNPs) [11], and show that the strains causing mass deaths were unrelated to either Medievalis or Orientalis biovars.

## Results

### Detection of *Y. pestis* markers in individuals from mass graves during the Black Death and the second pandemic

**Figure 1:**
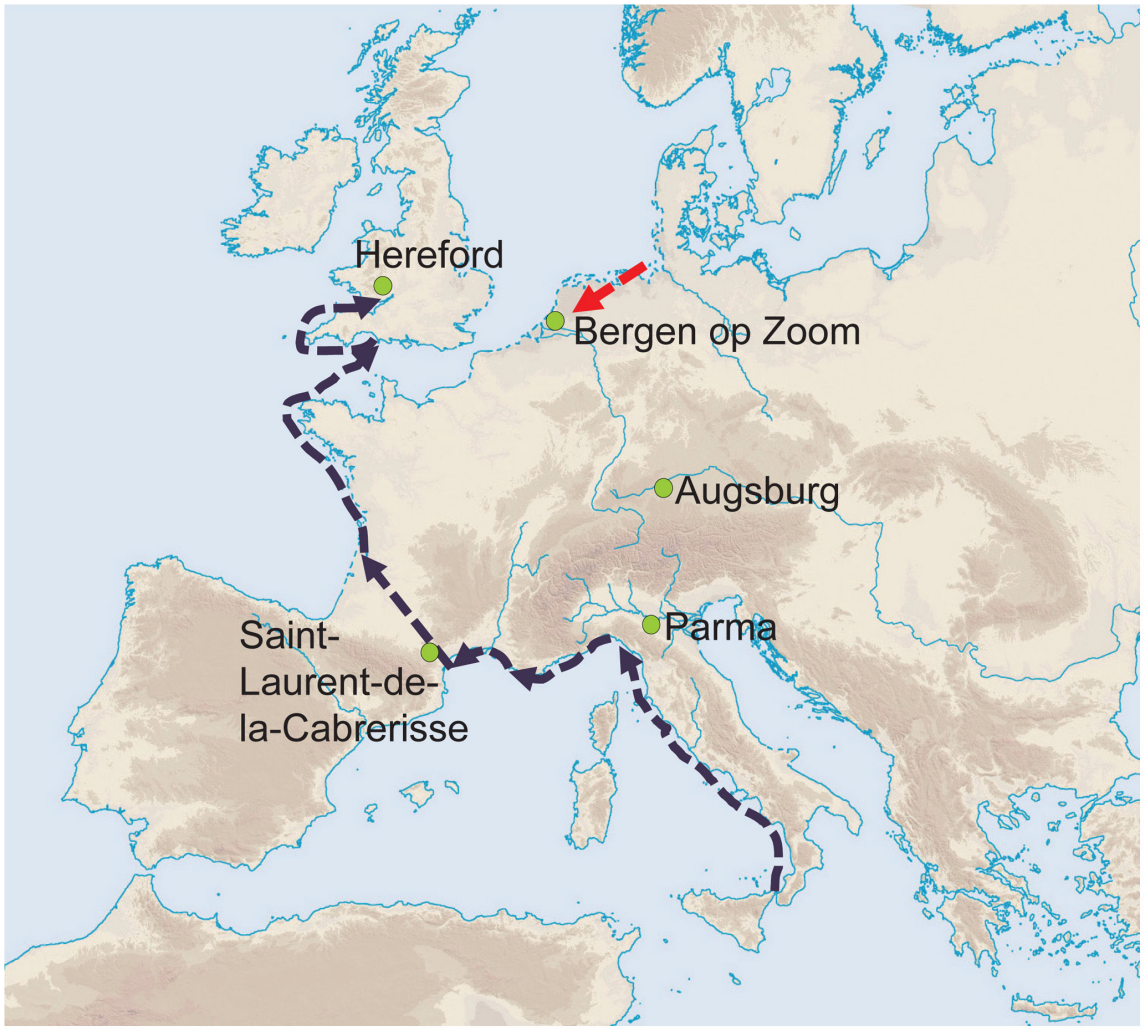
Geographical position of the five archaeological sites investigated. Green dots indicate the sites. Also indicated are two likely independent infection routes (black and red dotted arrows) for the spread of the Black Death (1347–1353) after Benedictow [25].

aDNA analyses were performed with dental pulp or bone samples from 76 human skeletons excavated from putative plague pits in England, France, Germany, Italy, and the Netherlands (Fig. 1). These “plague pits” were dated to the 14^th^–17^th^ centuries on the basis of ^14^C dating or archaeological evidence (Table 1 and Text S1). The samples were tested in a specialized aDNA laboratory using accepted precautions for avoiding DNA contamination and false positive results. PCR amplification was used to search for the presence of the *Y. pestis*-specific *pla* gene that is located on the multicopy plasmid pPst. This gene has been previously used to test plague skeletons dating from the 14^th^–15^th^ centuries, from 1722, as well as from Justinian's plague [7], [13]. We repeatedly obtained an amplification fragment of the expected size from ten individuals from France, England and the Netherlands (Table 1). Experimental contamination is unlikely because modern *Y. pestis* was never investigated in our laboratories, and we regularly used milling, extraction and amplification of blank samples to confirm the absence of contamination from the laboratory environment. Moreover, for each reaction set, several experimental samples failed to yield an amplification product, which also supports the absence of laboratory contamination. The absence of contamination from the burial environment is indicated by a soil sample from the vicinity of individual Man 30 (Table S3), which was found to contain no trace of *Y. pestis* DNA (also see Text S1). We also failed to amplify products from samples of 28 individuals that had been buried in the same cemeteries or localities as the suspected plague victims, but during periods where there was no archaeological indication of plague epidemics (Table 1), which is a further negative control (also see Text S1 and Table S4). Finally, a potential inhibition of PCR reactions from these 28 negative controls was excluded because we were successful in attempts to amplify human mitochondrial DNA sequences from the same samples.

**Table 1:** Human samples used for the detection of *Y. pestis* traces and results of genetic and immunochromatographic analyses.

Archaeological site (abbreviation)	Country	Dating#	Samples for aDNA	aDNA§			Samples for F1	F1-antigen§
				*pla*+	*caf1*+	*rpoB*+		RDT+
Bergen op Zoom (Ber)	the Netherlands	Mid-14^th^ C.(AD 1349-50?)	Teeth	7/43	3/7	2/4	Bones	3/5
Augsburg (Man)	Germany	16^th^/17^th^ C.	Bones (one tooth)	0/7	0/7	0/7	Bones	4/7
Parma (Par)	Italy	16^th^/17^th^ C.(AD 1629-30?)	Bones	0/4	0/8	0/8	Bones	6/19
Hereford (Her)	England	calAD 1335±54(KIA 23704)	Teeth	2/12	1/2	0/12	Teeth	4/7
Saint-Laurent-de-la-Cabrerisse (SLC)	France	AD 1348 or 1374?(OxA 21213-15)*	Teeth	1/6	1/1	n. t.	Bones	7/9
Bösfeld**	Germany	ca. 7^th^ C.	Bones	0/2	0/2	0/2	Bones	0/2
SLC-Neg.**	France	8^th^-10^th^ C.	Teeth	0/6	n.t.	n.t.	Bones	0/6
BNK **	the Netherlands	16^th^/17^th^ C	Teeth	0/20	0/2	n.t.	Bones	0/20

aDNA samples were screened by PCR and F1-antigen was detected by immunochromatography.

# Dating was determined on the basis of archaeological context (see Text S1) or radiocarbon dates. Question marks indicate the likely date for the archaeological site. Numbers represent Oxford (OxA) and Kiel (KIA) laboratory numbers for radiocarbon dates, calibrated in calAD using the program OxCal.

**§:** For each analysis the success rate is reported as a relative frequency based on the number of individuals analyzed. n.t.: not tested.

*See Text S1 for more details.

**Negative controls.

The DNA sequences of the amplified *pla* fragments were almost identical to those in extant *Y. pestis* strain CO92 (Fig. S1 in Text S2), except for a few inconsistent nucleotides, as is commonly observed in aDNA analyses due to *post-mortem* DNA degradation. We further investigated the *pla* positive samples by PCR amplification of a second *Y. pestis*-specific gene, *caf1*, which is carried on the low copy number pFra plasmid. We also attempted to amplify the chromosomal *rpoB* locus, which had previously failed to amplify in aDNA samples from various suspected European plague pits [21]. We were able to amplify the *caf1* locus from five *pla*-positive skeletal samples from the Netherlands, France and England (Table 1) and *rpoB* from two *pla*-positive individuals from the Netherlands. Again, the sequences of the aDNA gene fragments were almost identical to modern sequences from strain CO92 (Fig. S2 in Text S2).

None of the samples from Parma (Italy), or Augsburg (Germany) yielded PCR amplification products (Table 1). One possible explanation for these negative results is that we only had access to one tooth from the individuals buried in these two sites and all other materials that were tested consisted of bone samples, which probably contain lower levels of preserved aDNA than does tooth pulp. We therefore tested these individuals for plague by an alternative, protein-based method, namely immunochromatography by a dipstick test which has previously detected *Y. pestis*-specific F1 antigen in ancient human remains [9], [22], [23]. This method is well-suited for detecting plague in historical samples because proteins are more resistant to environmental degradation than is aDNA [8]. Immunochromatography confirmed the presence of the F1 antigen in bones and teeth from individuals at the three sites (Bergen op Zoom, Hereford and Saint-Laurent-de-la-Cabrerisse) where *Y. pestis*-specific aDNA had been detected in tooth pulp. Furthermore, samples from Parma and Augsburg that did not yield PCR products were positive for F1 antigen. Similar to the aDNA results, antigen was not detected in negative control samples or in soil samples from those sites (Tables 1 and S1).

In summary, two independent methods demonstrate that humans buried in mass graves that were historically and contextually associated with the Black Death and its resurgences, were consistently infected by *Y. pestis* in southern, central and northern Europe. Thus, the second pandemic was probably caused in large part by *Y. pestis*.

### Genotyping analyses

Although our results demonstrated the association of the plague bacillus with the second pandemic, they did not define the genotype of *Y. pestis* that was responsible for plague, nor did they define their genetic relationships to extant bacteria that continue to cause the disease today.

We tested whether the ancient strains that caused these massive deaths were of biovars Orientalis or Medievalis by testing the aDNA for characteristic mutations in *glpD* and *napA*. We were able to PCR amplify and sequence the *glpD* locus from 7 of 9 *pla*-positive individuals from the Netherlands, France and England. In contrast to prior results [14], none of the sequences contained the characteristic 93 bp *glpD* deletion associated with biovar Orientalis (Table S3). Similarly, the stop codon characteristic of Medievalis was absent in the *napA* amplified product from four *pla*-positive individuals from the Netherlands and England (Table S3). Therefore, the *Y. pestis* strains infecting those individuals were neither Orientalis nor Medievalis.

**Figure 2:**
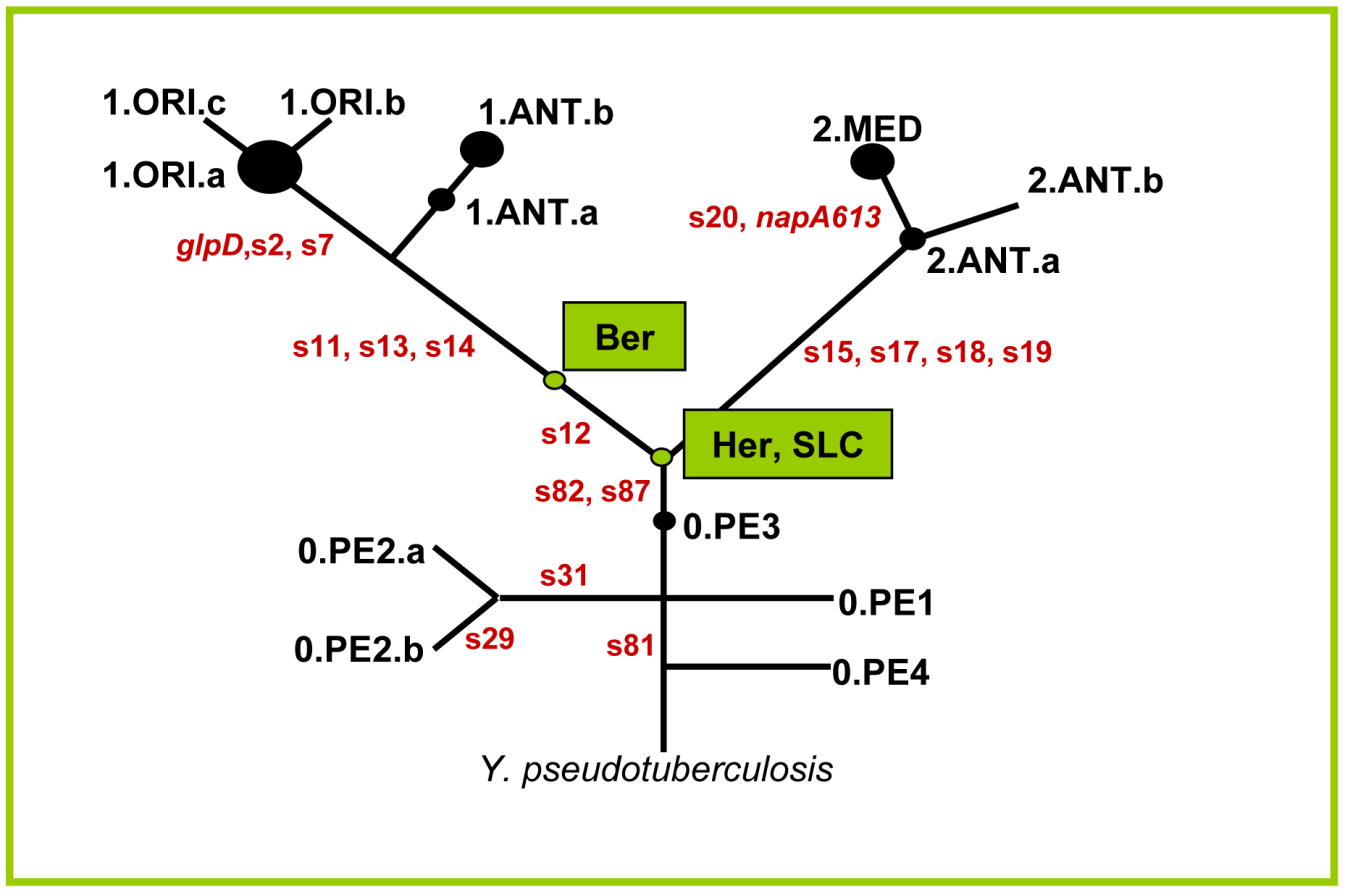
Schematic phylogenetic tree of *Y. pestis* derived from Achtman *et al.* [**11**] with the position of the ancient strains. The SNPs used to define the position of the ancient strains in the tree are indicated along each branch. The genotype from Hereford and Saint- Laurent-de-la-Cabrerisse is located at the node between branches 0, 1 and 2 because it had derived SNPs for branch 0, but only ancestral SNPs for branches 1 and 2. The genotype from Bergen op Zoom had one additional derived SNP (s12) on branch 1, but SNPs s11, s13 and s14 on branch 1 were ancestral.

**Figure 3:**
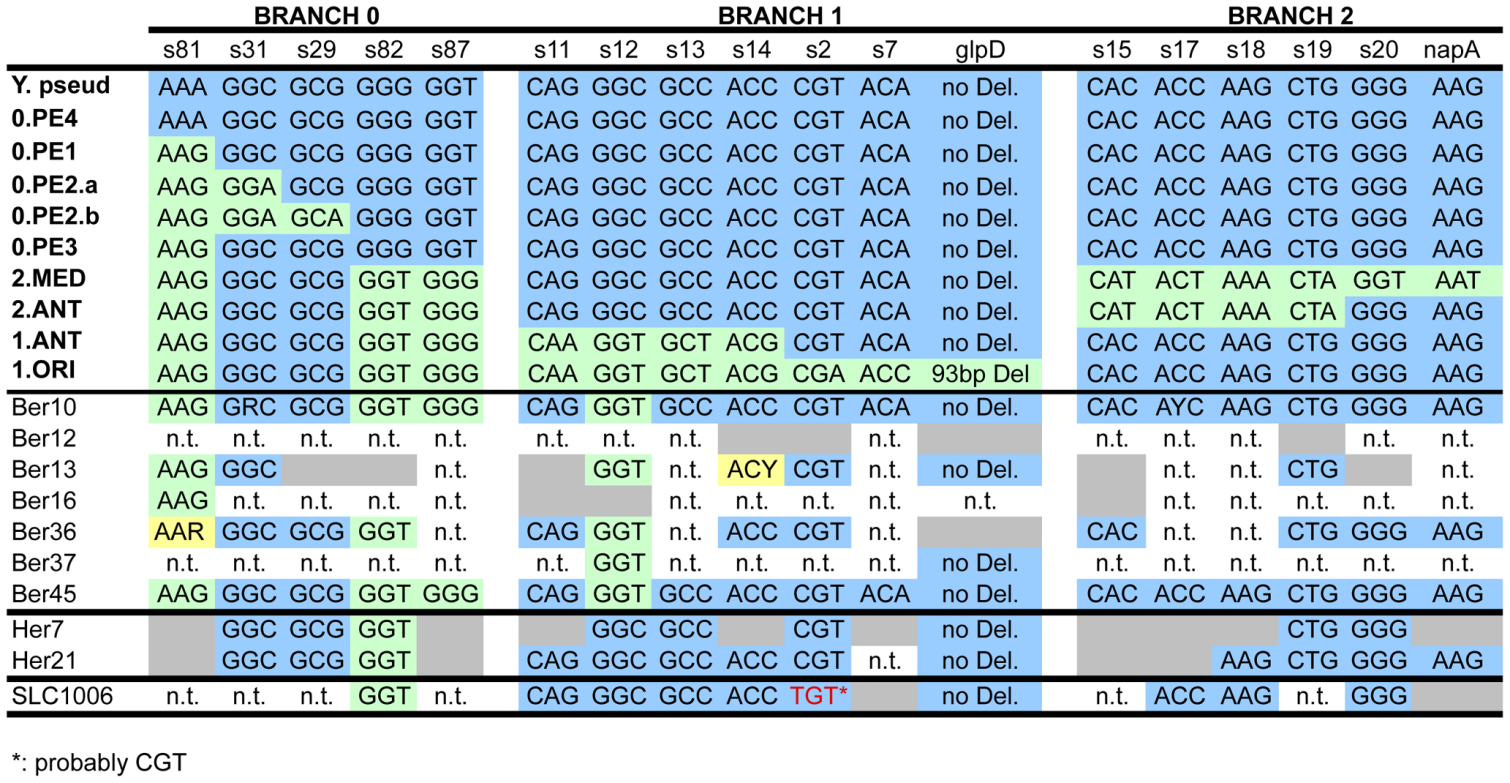
Analysis of aDNA from human remains with 18 markers (*glpD*, *napA* and 16 SNPs [**11**]) that define the three main branches of the *Y. pestis* evolutionary tree. The upper part shows the *Y. pestis* codons that were either similar to the *Y. pseudotuberculosis* ancestor strain (in blue) or exhibited a SNP (in green). These codons were used here to describe the various branches and sub-branches of the *Y. pestis* microevolutionary tree, as defined in [11]. The lower part of the figure shows the codons obtained at each specific position in the aDNA from individuals originating from three different archeological sites (Bergen op Zoom [Ber], Hereford [Her], and Saint-Laurent-de-la-Cabrerisse [SLC]). Samples which failed to give an amplification product are indicated in grey. Yellow indicates sequencing results that did not unambiguously resolve a SNP. n.t.: not tested. *: Probably CGT, which could give rise to TGT by deamination.

*Y. pestis* evolved from its parent species *Yersinia pseudotuberculosis* within the last 20,000 years [24]. A comparison of three genomic sequences plus other molecular markers positioned modern strains on a phylogenetic tree (Fig. 2) which consists of three major branches designated branches 0, 1 and 2 [11]. Most Orientalis isolates correspond to a monophyletic group designated 1.ORI, while most Medievalis isolates correspond to a second monophyletic group designated 2.MED. Antiqua strains are found in other populations on branch 1 (1.ANT) as well as branch 2 (2.ANT). Still other *Y. pestis* strains, known as biovar Pestoides, cluster in various populations along branch 0 (0.PE1-0.PE4). We attempted to identify the genotypes causing the second plague pandemic in our samples by testing 16 characteristic synonymous single nucleotide polymorphisms (SNPs) that mark major phylogenetic branch points within this *Y. pestis* evolutionary tree (Fig. 3). These SNPs were amplified and sequenced from *pla*-positive individuals from whom sufficient biological material was available (Fig. 3, Table S3). Again, only limited nucleotide diversity attributable to DNA degradation was found, and the nucleotides at the targeted SNP positions could be unambiguously and reproducibly determined in six plague victims from the Netherlands, two from England and one from France (Fig. 3; Fig.s S4, S5 and S6 in Text S2). The results confirmed that the genotypes of *Y. pestis* identified in our archaeological samples from the 14^th^ century are distinct from modern Orientalis (1.ORI) and Medievalis (2.MED), and showed that they also differ from modern Antiqua (1.ANT, 2.ANT) (Fig.s 2 and 3). Instead, the genotypes map to that part of the phylogenetic tree where branches 0, 1 and 2 separate. These genotypes have evolved more recently than populations 0.PE1 through 0.PE4 on branch 0 because they have derived versions of SNPs s81, s82 and s87. However, they evolved earlier than either 1.ANT or 2.ANT because they have ancestral versions of SNPs s11, s13, s14, s15, s17, s18 and s19 (Fig. 3). The samples from France and England also have the ancestral version of SNP s12, which places them near the split between branches 0, 1 and 2. In contrast, the samples from the Netherlands have the derived version of SNP s12, which maps them to the beginning of branch 1.

All the ancient samples are thus distinct from modern *Y. pestis* from Africa, the Americas, and the Near East, as well as the Pestoides isolates from the former Soviet Union that have been tested [11]. These results also indicate that at least two related but distinct genotypes of *Y. pestis* were responsible for the Black Death and suggest that distinct bacterial populations spread throughout Europe in the 14^th^ century.

## Discussion

From historical accounts we know that the Black Death was imported into southern Europe from Asia, and reached Marseilles (southeast France) by November 1347. Plague then spread to western France by land and sea, reaching Narbonne and Carcassonne at the beginning of 1348 [17], [25]. This extension to the west was probably responsible for a plague epidemic in Saint-Laurent-de-la-Cabrerisse, which lies between Narbonne and Carcassonne. Although written records are not available to confirm that supposition, radiocarbon measurements on skeletons from the three multiple graves in Saint-Laurent-de-la-Cabrerisse date those skeletons to the outbreak of AD 1348 or 1374. Plague continued to spread to northern France in early 1348, and is thought to have been exported from France to England via shipping and trading in the summer of that year [26]. Hereford, a busy English market town near the Welsh border, and a centre for religious pilgrimage, recorded its first plague deaths in the spring of 1349, followed by a second outbreak in 1361, and a third in 1369 [27]. The Hereford plague pits have been AMS radiocarbon dated to calAD 1335±54 and our specimens can therefore be attributed to one of these three epidemic waves. Our finding of identical genotypes (based on 20 markers) in Saint-Laurent-de-la-Cabrerisse and Hereford thus lends support to historical evidence [2], [25] which suggest that plague spread from France to England (Fig. 1) in the second half of the 14^th^ century.

Bergen op Zoom was a thriving port city in the south of the Low Countries. There are no written reports describing plague in Bergen op Zoom for the years 1348–1351 or in subsequent decades because administrative records were destroyed by fire in 1397. Radiocarbon dating has not been performed on the skeletal remains of the circa 800 individuals buried in the mass graves from Bergen op Zoom, but soil stratigraphy, artifacts and coins allow dating to the mid-14^th^ century. Historical records indicate that the Black Death reached the southern Low Countries from France or England in 1349 and that the northern Low Countries were infected from Friesland in 1350 [25]. However, the *Y. pestis* genotype identified in our skeletons from Bergen op Zoom differed from those found in Hereford and Saint-Laurent-de-la-Cabrerisse, implying that Bergen op Zoom (and possibly other parts of the southern Netherlands) was not directly infected from England or France in AD 1349. Instead, our results are more consistent with the idea that the genotype in Bergen op Zoom represents a different route of plague spread, possibly from the northern to the southern Low Countries in AD 1350 (Fig. 1). Bergen op Zoom was in intensive commercial contact with the north of the Netherlands in the 14^th^ century, exporting pottery to Amsterdam [28]. Friesland, in turn, was infected from Norway, which during the Middle Ages traded extensively with both England and the Hanseatic cities along the North Sea coast of Germany.

Only one SNP, s12, was identified that distinguished the genotypes in Bergen op Zoom and Hereford, indicating that they are closely related. That single SNP is highly informative because it is on branch 1, showing that the genotype found in Bergen op Zoom evolved on the phylogenetic path to 1.ORI, the causative agent of the third pandemic that erupted in Hong Kong in 1894. If SNP s12 had evolved anywhere within Europe, then *Y. pestis* must have subsequently spread from Europe to Central and East Asia, the source of the third pandemic associated with 1.ORI [11]. Alternatively, and probably more likely, multiple genotypes were imported to Europe via the usual routes of trade from Central Asia during the 14^th^ century and afterwards. In that event, the lineage from Bergen op Zoom could represent a distinct wave of infection relative to those from Hereford and Saint-Laurent-de-la-Cabrerisse that reached the Low Countries from Norway, the Hanseatic cities, or another site that is not intuitively obvious from the historical records.

Together with prior analyses from the south of France [5], [6] and Germany [7], our data from widely distributed mass plague pits ends the debate about the etiology of the Black Death, and unambiguously demonstrates that *Y. pestis* was the causative agent of the epidemic plague that devastated Europe during the Middle Ages. However, there is an apparent discrepancy between the genotypes indentified in this study and those reported elsewhere on aDNA from Justinian's plague and the second pandemic [13], [14]. In the first of these analyses, aDNA from Justinian's plague and the second pandemic was concluded to correspond to biovar Orientalis (now designated 1.ORI) on the basis of microsatellites. That assignment is not definitive because only isolates of four modern populations (1.ORI, 2.MED, 1.ANT and 2.ANT) were used for comparative purposes, and it is not clear whether microsatellites could distinguish 1.ORI from other historical populations on branch 1 [29]. The second analysis demonstrated the presence of the 96 bp *glpD* deletion that is characteristic of 1.ORI in samples from the 7^th^ to 9^th^ century (Justinian's plague) and about 1720 (the end of the second pandemic). In our study we did not find the *glpD* deletion in *Y. pestis* aDNA from the Black Death period. We did not test aDNA from the same sources and dates, and the possibility that 1.ORI bacteria were imported from East Asia both before and after the Black Death cannot be excluded. However, new data suggesting that 1.ORI evolved in the last 212 years [30] argue against this possibility.

The ancient genotype from Bergen op Zoom described here differs from all known modern populations from three continents [30], and might now be extinct. The strains from France or England may still exist because the SNPs that have currently been tested match the genotype of isolates from China that have recently been assigned to a new branch, designated branch 3 [30]. Additional SNPs that are specific for branch 3 will first need to be identified and tested to determine whether the ancient populations from France and England matches existing isolates. These tests should involve further genotyping of large, global collections of extant lineages in combination with extensive palaeogenetic analyses of other mass graves from the historical routes travelled by the Black Death and Justinian's plague. Such analyses are very promising because they could potentially fully reconstruct the history of ancient plague pandemics within the modern phylogeographical context of *Y. pestis*.

## Materials and Methods

### Samples

For aDNA analyses, one or more teeth or bone samples were taken from 76 presumed plague victims from various locations in Europe (Table 1). Archaeological information on the single plague sites can be found in more detailed form in Text S1. Teeth were preferred as sample material due to better aDNA preservation, and where possible, two different teeth per individual were submitted for genetic investigation. If no teeth were available, femoral bone samples were used. Negative controls were obtained from individuals who either died before or after the Black Death, in a context that did not involve a major epidemic.

### Sample preparation and DNA extraction

aDNA analyses were carried out in the laboratories of the Institute of Anthropology in Mainz, Germany. The pre-PCR laboratories are situated in a humanities building physically separated from the post-PCR area, have dedicated equipment and are subjected to overnight UV-light exposure. Other anti-contamination measures used are extensively reported in previous publications [31], [32].

After arrival in the laboratory, samples were submitted to decontamination procedures consisting of 45 minutes of UV irradiation on each side, mechanical removal of the outer surface by sandblasting (Harnisch und Rieth, Winterbach, Germany) and a second UV irradiation. The samples were then fine powdered using a mixer mill (Retsch, Haan, Germany) and stored at 4°C or −20°C until use.

Aliquots of 0.38–0.5 g powder were incubated overnight on a rotary mixer at 37°C in a decalcification and digestion solution consisting of 0.5 M EDTA (pH 8; Roth, Karlsruhe, Germany), 0.4% N-Laurylsarcosine and 0.46 mg/ml Proteinase K (Roche, Germany). Post-digestion, DNA was extracted using phenol/chloroform/isoamyl alcohol (25∶24∶1 Roth, Karlsruhe, Germany) and trichlormethan/chloroform (ROTIPURAN ≥99%, p.a.; Roth, Germany); then desalted and concentrated using micro-concentrators (Centricons 100, 50 or Amicon Ultra-15 Centrifugal Filter Units, Millipore, Schwalbach/Ts., Germany). When possible, several independent extracts were obtained from two or more different samples from each individual (see Table S3). In addition to blank extraction controls, a negative control consisting of hydroxyl apatite was generally co-processed by milling, and further co-extracted and co-amplified throughout the analyses (milling blank). Sterile aliquot reagents were changed frequently.

To monitor the bacterial content of the soil, 500 mg of earth associated with the sample Man30 (Augsburg) was analyzed. The sample was incubated in 1 ml buffer consisting of 100 mM NaCl, 10 mM Tris/HCl (pH 8), 50 mM EDTA (pH 8) and 0.5% SDS and submitted to 20 minutes sonication (EMMI 30HC, EMAG Technologies, Germany) at 50°C. Incubation on a rotary mixer overnight at 37°C followed after adding 0.1 mg/ml Proteinase K. DNA extraction was carried out with phenol/chloroform/isoamyl alcohol as described above. For desalting and concentration, both Centricons 50 and Amicon Ultra-15 Centrifugal Filter Units were used.

### Amplification of *Y. pestis* specific genes

The amplifications of the *Y. pestis* specific genes were performed using the primers described in Table S2. The amplification reaction was set up with 2–10 µl of extracts in a final volume of 50 µL, 1–1,2× PCR Gold Buffer (Applied Biosystems, Darmstadt, Germany), 2.5 U Ampli-TaqGold (Applied Biosystems, Darmstadt, Germany), 2.5–3 mM MgCl_2_ (Applied Biosystems), 0.2 mM dNTP mix (Qiagen GmbH, Hilden, Germany), 0.2 µM each Primer (Biospring, Frankfurt am Main, Germany), 8 µg/ml BSA (Roche) and UV irradiated HPLC water (Arcos Organics/Fisher Scientific, Germany). The amplifications were carried out in a Mastercycler Gradient (Eppendorf, Hamburg, Germany). The cycle program consisted of an initial denaturation at 94°C for 6 minutes, followed by 50 cycles of 40 seconds at 94°C, 40 seconds at 52–62°C and 40 seconds at 72°C. In the different PCR sets, samples were co-processed together with milling, extraction and amplification controls.

Amplification of the soil sample was performed with two different concentrations of the target (1∶1 and 1∶50, each time 0.1–5 µL target) using the primer pairs 16S F3/R3 and rpoB F1/R1 as described above.

### Sequencing

Amplification products were purified using the Invisorb Rapid PCR Purification Kit (Invitek, Berlin-Buch, Germany) according to the manufacturer's instructions. Alternatively, the purification was carried out by digestion with *Exo*I (20 U/µL; Fermentas, St. Leon-Rot, Germany)/SAP (1 U/µL; Fermentas, St. Leon-Rot, Germany) enzymes by incubating the reaction mix for 45–60 minutes at 37°C. The successive enzyme inactivation at 80°C for 15 minutes concluded the procedure.

Sequencing of successfully amplified fragments was carried out with the DNA Sequencing Kit (BigDye Terminator v3.1 Cycle Sequencing Kit; Applied Biosystems) using 25 cycles at 92°C for 30 seconds, 15 seconds at 52–62°C and 2.5–3 minutes at 60°C. Cycle sequencing products were purified by using Sephadex-G50 Fine (GE Healthcare, Uppsala, Sweden) and analyzed by capillary electrophoresis on ABI PRISM 3130 Genetic Analyzers (ABI PRISM Applied Biosystems).

### Sequence analyses and alignments

The sequences were further analyzed using the program Seqman II and MegAlign from the DNA Star software package (version 7.0.0). The sequence alignments are listed in Text S2 (Fig.s S1, S2, S3, S4, S5 and S6). The reference sequences used to align the sequenced products are listed below (paragraph ‘Accession numbers’). Strain CO92 belongs to branch 1.ORI and strain KIM belongs to branch 2.MED according to the proposed classification [11]. The position of the single SNPs was previously published [11].

### Rapid diagnostic test (RDT)

Nineteen samples from Parma, seven samples from Augsburg, five samples from Bergen op Zoom, seven samples from Hereford and nine from Saint-Laurent-de-la-Cabrerisse were tested along with soil samples and 28 negative controls with double blind procedures. The analyses were carried out at the Laboratory of Parasitology and Parasitic Diseases, Department of Animal, Production, Epidemiology and Ecology at the University of Turin, Italy. Spongy bone from femora was chosen as a preferential source of material [22]. The bone samples were cleaned with dry brushes and decontaminated by UV light. The external bone surface was removed with a drill (Kavo Intramatic Lux 2) while mounted on a micromotor turning at 9,000 rpm. Next, the spongy bone samples (1–2 g) were powdered by hand in sterile conditions using a mortar and pestle. Powder from each sample was stored in 15 ml sterile vials until use. Tooth samples were prepared as described for aDNA analyses.

To detect *Yersinia pestis* F1 antigen, we used the plague dipstick assay developed and tested on extant sufferers by the Institute Pasteur of Madagascar and Paris (rapid diagnostic test for plague- RDT). This immunochromatographic-based assay detects the F1 envelope glycoprotein specific to *Y. pestis* [33], [34]. The principles of the dipstick assay and methods for semi-quantitation of the AgF1 concentrations in ancient specimens have been described elsewhere [9]. For each sample, extracts were prepared from 50 mg of bone material, tooth or soil samples reconstituted in 200 µl of sterile saline solution and subjected to the following protocol: three freeze/thaw cycles, sonication for 15 minutes, and a fourth freeze/thaw cycle. The suspensions were incubated for 24 hours at 4°C to solubilize the remaining antigens. The crude extracts were then centrifuged at 5,000 rpm at room temperature and the supernatant submitted to dipstick assay. The tests were repeated five times on the same specimen and the results were read after 15 minutes. The detection threshold of the test (0.5 ng/ml) is diagnostic for *Y. pestis* infection in ancient skeletal remains [9], [22], [23]. The results of the investigation are detailed in Table S1.

### Accession numbers

The GenBank (http://www.ncbi.nlm.nih.gov) accession numbers for DNA sequences longer than 50 bp determined in this paper are HM752036-HM752097. All alignments are published in Text S2 (Fig.s S1, S2, S3 S4, S5 and S6). To align sequenced products, the reference sequences from *Y. pestis* CO92 (NC_003134; AL590842), *Y. pestis* KIM (AE009952.1) and *Y. pseudotuberculosis* IP32953 (BX936398) were used for genomic markers and SNPs, whereas *caf1*- and *pla*-sequences were aligned with NC_003132 (AL109969) and NC_003134 (AL117211).

## Supporting Information

Text S1 Detailed archaeological and genetical information
(0.08 MB DOC)

Text S2 Supplementary figures S1 to S6 of all aligned sequences
(0.84 MB DOC)

Table S1 Results of the RDT analyses
(0.10 MB DOC)

Table S2 Primers used in this study
(0.09 MB DOC)

Table S3 Amplified products obtained from the various samples analysed, using different sets of primers
(0.86 MB DOC)

Table S4 Summary of the results and test of the hypothesis of false negatives among the negative controls
(0.03 MB DOC)
