# Supplementary material for: Distinct Clones of Yersinia pestis Caused the Black Death
Source: PLoS Pathog. 2010 Oct 7;6(10):e1001134. doi: 10.1371/journal.ppat.1001134 (PMC2951374; doi:10.1371/journal.ppat.1001134)
Supplement: Text S1 — Detailed archaeological and genetical information (0.08 MB DOC) [file ppat.1001134.s001.doc]

**Distinct clones of *Yersinia pestis* caused the Black Death**

Haensch S, Bianucci R, Signoli M, Rajerison M, Schultz M, Kacki S, Vermunt M, Weston DA, Hurst D, Achtman M, Carniel E, Bramanti B.*

*To whom correspondence should be addressed at the Institute of Anthropology,

Colonel Kleinmann Weg 2, SBII 02, Johannes Gutenberg University,

D-55128 Mainz, Germany. E-mail: bramanti@uni-mainz.de

**Archaeological information**

The rural cemetery of Saint-Laurent-de-la-Cabrerisse is located mid-way between Narbonne and Carcassonne in south-eastern France. During the 2007 excavations, an early medieval church and graveyard used during the 8^th^-14^th^ centuries were discovered and 149 graves were identified. Three of the graves, located close to the southern wall of the church, were mass graves belonging to a funerary phase dating between the 11^th^ and 14^th^ centuries. Two of these interments contained two bodies each, while the third contained the remains of five individuals, making a total sum of six adults and three juveniles. As all the bodies were buried simultaneously and there were no signs of violent trauma, the three multiple graves were suspected to be plague pits. Historical accounts suggest that the Black Death reached Marseilles (in south-eastern France) by November 1347 and spread to the west of France by land and sea, reaching Narbonne and Carcassonne at the beginning of 1348 [S1, S2]. Unfortunately, no additional written records exist for the rural cemetery of Saint-Laurent-de-la-Cabrerisse. A copper and iron buckle found in association with one skeleton (SLC 1083) from grave SLC 160, together with soil stratigraphy dated the graves to between the early 13^th^ and late 14^th^ centuries, indicating that the plague epidemic associated with these graves could be either the outbreak of 1348 or the resurgence of 1374. Radiocarbon measurements have been carried out on three samples from the site, among these one from grave SLC 141, the grave of the PCR-positive sample SLC 1006 (sample SLC 1010; OxA-21213). For this sample, calibration indicates calAD 1279-1389 (95.4 % probability) as the most likely range, i.e. calAD 1279-1315 (49.6 %) and 1355-1389 (46.4 %). Individuals from the two other pits, which were positive only to the RDT analyses, were also dated: SLC 1013 (OxA 21214) - calAD 1286-1325 (39.7 %), 1345-1394 (55.7 %); SLC 1081 (OxA 21215) - cal AD 1303-1366 (70.7 %), 1383-1409 (24.7 %).

From the English cemetery site of Hereford, a town near the Welsh border, 188 individuals were found in three mass graves (pits) associated with the cathedral [S3]. The pit sample, a relatively small proportion of the 1194 skeletons excavated from the cemetery, had a large number of juveniles, particularly children aged between 5 and 14 years. Adults from the mass pits were mainly aged between 26-35 years, and the sexes were evenly represented. The three pits were of similar dimensions and were found in a row, each built up of layers containing bodies separated by thin deposits of clay-rich soil. It is likely that many more individuals (ca. 300-400) were buried in the mass graves, as only the end of each burial pit was excavated. All archaeological evidence points to the Black Death as the cause of death for the individuals in the mass graves. Plague is thought to have been exported from France to England via shipping and trade in the summer of 1348 [S4]. Most of the Hereford samples used here were derived from individuals from Plague Pit 2, which has been directly AMS radiocarbon dated to between calAD 1281-1389 (2 sigma range, probability 95.4 %, KIA23704), and fits into either the time of the first plague outbreak in 1349, the second outbreak in 1361, or the third outbreak in 1369 [S5].

The skeletal collection from Bergen op Zoom (The Netherlands) was excavated from the site of an ancient hospital, where all the available ground was used for mass burial. The samples were taken from eleven mass graves containing between 10 and 25 individuals, most of them buried in wooden coffins, some in linen shrouds. The mass graves, which can be dated to the middle of the 14^th^ century on the basis of soil stratigraphy, artefacts and coins, were located very close to one another, did not intersect and were all contained within the same stratigraphic level. Moreover, 30 graves were found surrounding the altar inside the hospital chapel and an additional 150 graves were found in the same level as the central hospital building itself and in a small structure adjacent to the hospital [S6]. Out of an estimated population of 2,400-3,200 medieval inhabitants, approximately 800 were buried in the mass graves, indicating a sudden, high mortality due to a major catastrophe. The administrative records for Bergen op Zoom, once a thriving port city, were destroyed by fire in 1397 and there are no written reports describing plague for the years 1349-1351 or later. However, historical data indicate that plague ravaged the Low Countries at least four times between 1349 and 1390.

Plague epidemics have been well-documented in the southern German town of Augsburg since 1380, repeatedly afflicting the town until 1634/1635. During the 15^th^ and 16^th^ centuries, plague episodes were recorded once a year, and in the years 1627/1628 a total of 12,103 people died [S7]. The excavated site (now factory premises at Heinrich-von-Buz-Straße 28) was dated to the 16^th^/17^th^ century by ceramics and was associated with either the Thirty Years War or a disease epidemic [S8]. This mass grave was found roughly 300m away from a site excavated in 1936 near an earlier plague repository. The mass grave consisted of 4-5 layers of skeletons, which were difficult to separate. The individuals were all buried together in an anonymous fashion and all but 9 individuals were placed in a prone position, alternating east-west, reflecting the typical rapid burial of plague victims.

The Italian archaeological site of Ex-Gelmini in Parma was situated in the Oltretorrente area of the city, where several lazarettos (quarantine stations) were organized during the plague epidemic of 1630 [S9]. During the excavation, numerous pits were discovered and estimated to contain up to 5,000 individuals in several stratified layers. Associated ceramics date the pits to the end of the 16^th^ or beginning of 17^th^ century, indicating that the skeletons might belong to victims of the 1630 plague wave that killed 75 % of the population (ca. 16,000-18,000 individuals [S10]).

The samples used as negative controls for the analyses (Table 1) originated from Germany, France and the Netherlands. Those indicated as ‘Bösfeld’ (Bös) were bones derived from skeletons excavated from the Bösfeld site, near Mannheim, a Frankish cemetery dating roughly to the 7^th^ century. The ‘SLC’ samples (all teeth) were unearthed from the oldest part of the cemetery of Saint-Laurent-de-la-Cabrerisse (8^th^-10^th^ centuries), whereas the teeth indicated as ‘BNK’ originated from sepultures in an ordinary cemetery of the 16^th^ and 17^th^ century in Bergen op Zoom. All samples used as negative controls were exhumed from sites without archaeological evidence of catastrophic events, like an epidemic. A soil sample for aDNA analysis was obtained from the femur of individual Man 30 from the Augsburg site.

**Additional considerations on the aDNA analysis**

For aDNA amplification we used oligonucleotide primers that were designed to amplify amplicons of 120 bp on average, a length that is compatible with degraded DNA (Table S2). Nevertheless, we failed to amplify the aDNA of *Y. pestis* from plague skeletons in 87 % of all individuals, similar to previous results obtained by other groups [S11, S12]. The recovery of amplifiable *Y. pestis* DNA from ancient samples is problematic even when analyses are repeated several times and the skeletons are from known plague victims. In some cases, only a few regions of the bacterial DNA could be amplified (Fig. 3; Table S3), probably because the DNA molecules were severely damaged and fragmented. For some individuals, even extracts obtained from different teeth from the same jaw showed differences in the success rate (*e.g.* Ber10, Table S3). Moreover, individuals who were positive for the RDT analysis often failed to amplify the *caf1* gene (Table 1), confirming published results by others [S12]. We did not consider two samples (Ber3 and Ber9) among the *pla*-positives, as although both amplified once, the amplification could not be positively replicated, even when the same extract was used. Most likely the DNA content in the extracts was too low and thus the possibility for amplification was strongly influenced by chance. However, Ber3 yielded positive results for the RDT analysis.

With regards to the series that failed to amplify *Y. pestis* specific DNA but were positive for the RDT reaction (Parma and Augsburg), we stress that for these two series bone samples were principally analyzed. While trabecular bone from the femoral heads revealed positive results for the RDT test, the genetically analyzed cortical bone may have possessed too few surfaces for bacteria to infiltrate in the last phase of infection. Moreover, unlike teeth, bone is more readily subjected to environmental degradation processes that destroy DNA.

For those samples that were positively amplified, all the sequences obtained were determined by up to three independent extractions and several independent amplifications, depending on the availability of biological material and extract (Table S3). When necessary, amplifications were repeated, changing the amount of template to exclude artifacts like stochastic substitution due to degradation processes, especially if those were observed at the SNP positions.

Each obtained sequence was tested by performing BLAST searches to find matches from *Yersinia* sequences sharing a MaxIdent score over 90 % (identity scores lower than 100 % were due to deaminations since the largest scores still remained *Y. pestis* ssp.). Deaminations are the principal changes that occur due to the degradation of ancient DNA [S13], thus suggesting an archaic origin for the molecules.

**Blanks and Negative controls**

No milling, extraction and PCR blanks amplified with *Y. pestis* specific primers yielded amplification results. An exception was a PCR control amplified for the SNP s19. This yielded a sequence that could be identified as *Escherichia coli* DNA (Fig. S6 in Text S2). The presence of traces of *E. coli* DNA in the laboratory environment is not unexpected since chemicals employed for the PCR reaction can be obtained from the modified bacterium. Blank controls amplified with unspecific primers (*rpoB* and 16S primers) in some cases gave reaction products that could always be unambiguously identified with primer dimers or artifacts.

Soil samples used for the RDT analyses always yielded negative results. Soil from one Augsburg bone sample (Man30) was used for genetic analyses. While *rpoB*-primers yielded no amplification, DNA amplified with the 16S-primers yielded only DNA from environmental and soil bacteria and no *Y. pestis*-specific sequence could be obtained.

No reaction products were obtained from the negative controls used for the RDT and PCR analyses. This cannot be attributed to inhibition effects since from all negative controls (but Bös 844) human mtDNA sequences were amplified (data not shown).

The high number of likely false negatives among the samples from the plague pits both for RDT and PCR analyses required a large number of negative controls to ensure that the sequences and the protein did not stem from other unknown bacteria. The main goal of our aDNA investigation was to genotype ancient *Y. pestis* DNA, not to genetically prove the existence of the infection in each skeleton. Thus we concentrated our efforts on obtaining reproductions of up to 20 specific markers from some individuals (Table S3) instead of performing a clinical case-control experiment by repeating the analysis a fixed number of times. Conversely, in several cases we abandoned the search after the first attempts, conscious that in these cases we would have had more false negatives among the plague individuals. Nevertheless, to establish whether we used an adequate number of negative controls we performed a Fisher's exact test on a contingency table (Table S4) under the hypothesis that ‘case’ and ‘controls’ were individuals originating from the same population. This gave a high significant *p*-value for both the RDT (*P* 0.01 %) and PCR (*P* 2.25 %) analyses. This indicates that it is extremely unlikely that the negative results obtained from the negative controls (non-plague individuals) were due to false negative reactions.

**References**

S1. Biraben J-N (1975) Les hommes et la peste en France et dans les pays Européens et Méditerranéens, 2 vols. Paris: Mouton.

S2. Benedictow OJ (2004) The Black Death 1346-1353: the complete history. Woodbridge: Boydell Press.

S3. Weston DA, Boylston AE, Ogden AR, Hurst D (2007) The palaeodemography of the Black Death: the Hereford Cathedral Close cemetery. Am J Phys Anthropol S44: 247-248.

S4. Shrewsbury JFD (1970) A history of bubonic plague in the British Isles. Cambridge: Cambridge Univ. Press.

S5. Hatcher J (1977) Plague, population and the English economy 1348-1530. London: Macmillan.

S6. Vermunt M (2007). In: Degraeve A, Demeter S, De Meulemeester J, editors. Hospitalen van de Middeleeuwen en de moderne tijden. Brussels: Ministerie van het Brussels Hoodstedelijk Gewest, pp. 127-142.

S7. Grünsteudel G, editor (1998) Augsburger Stadtlexikon. Augsburg: Perlach-Verlag.

S8. Bakker L (1988) Zeitschrift des Historischen Vereins für Schwaben 81: 7.

S9. Banzola MO (1980) L’Ospedale vecchio di Parma. Notizie storiche e vicende costruttive precedute da una sintesi della formazione urbana di Parma, cenni sulle origini e sulla storia degli ospedali nell’Occidente elementi di storia dell’ospitalità a Parma. Parma: Palatina.

S10. Del Panta L, Livi Bacci M, Pinto G, Sonnino E editors (1996) La popolazione Italiana dal Medioevo a Oggi. Bari: Laterza.

**S11.** Gilbert MT, Cuccui J, White W, Lynnerup N, Titball RW *et al.* (2004) Absence of *Yersinia pestis*-specific DNA in human teeth from five European excavations of putative plague victims. Microbiology 150: 341-354.

S12. Pusch CM, Rahalison L, Blin N, Graeme JN, Czarnetzki A (2004) Yersinial F1 antigen and the cause of Black Death. Lancet Infect Dis 4: 484-485.

S13. Hofreiter M, Jaenicke V, Serre D, von Haeseler A, Pääbo S (2001) DNA sequences from multiple amplifications reveal artifacts induced by cytosine deamination in ancient DNA. Nucleic Acids Res*.* 29: 4793-4799.
