# Supplementary material for: Distinct Clones of Yersinia pestis Caused the Black Death
Source: PLoS Pathog. 2010 Oct 7;6(10):e1001134. doi: 10.1371/journal.ppat.1001134 (PMC2951374; doi:10.1371/journal.ppat.1001134)

Figure S1: Alignment of the *pla*-nucleotide sequences from ancient samples. As reference the sequence from *Y. pestis* CO92 was used (AL10996; 1.ORI). Sample Ber16 is not considered in the alignment because it was not sequenced.


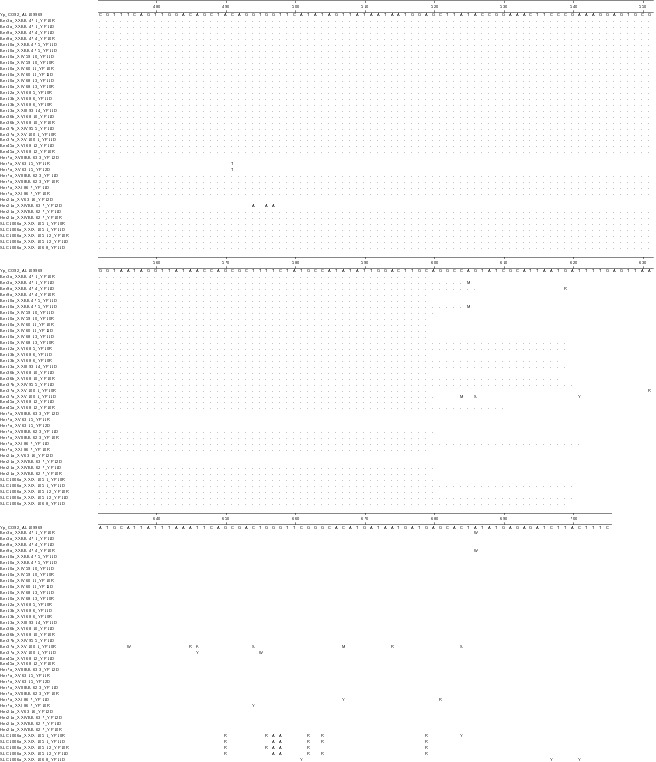


Figure S2: Alignment of the (A-B) *caf1* and (C-D) *rpo*B sequences from ancient samples. The corresponding gene sequences of *Y. pestis* CO92 served as reference sequence. Different primer sets were used for each amplification fragment (Tab. S2): for *caf 1* (A and B) caf1 F1/R1or U2 / L2 and rpoB F1/ R1 or rpoB F2 / R2 for *rpoB*.


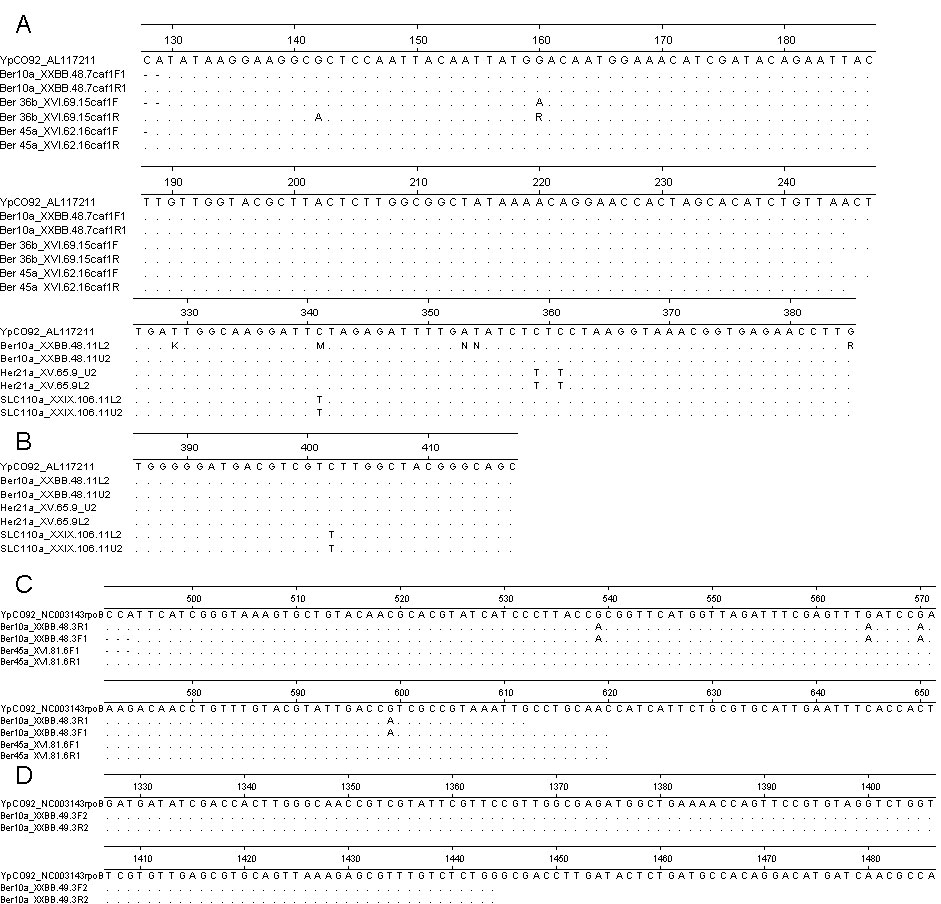


Figure S3: Sequence comparison of *glp*D (A) and *nap*A613 (B) of *Y. pestis* CO92 and KIM6 with sequences from ancient samples. Ancient samples lack both, the 93bp-deletion of 1.ORI and the G/T mutation of 2.Med (indicated with red boxes in S3.A and S3.B, respectively).

*
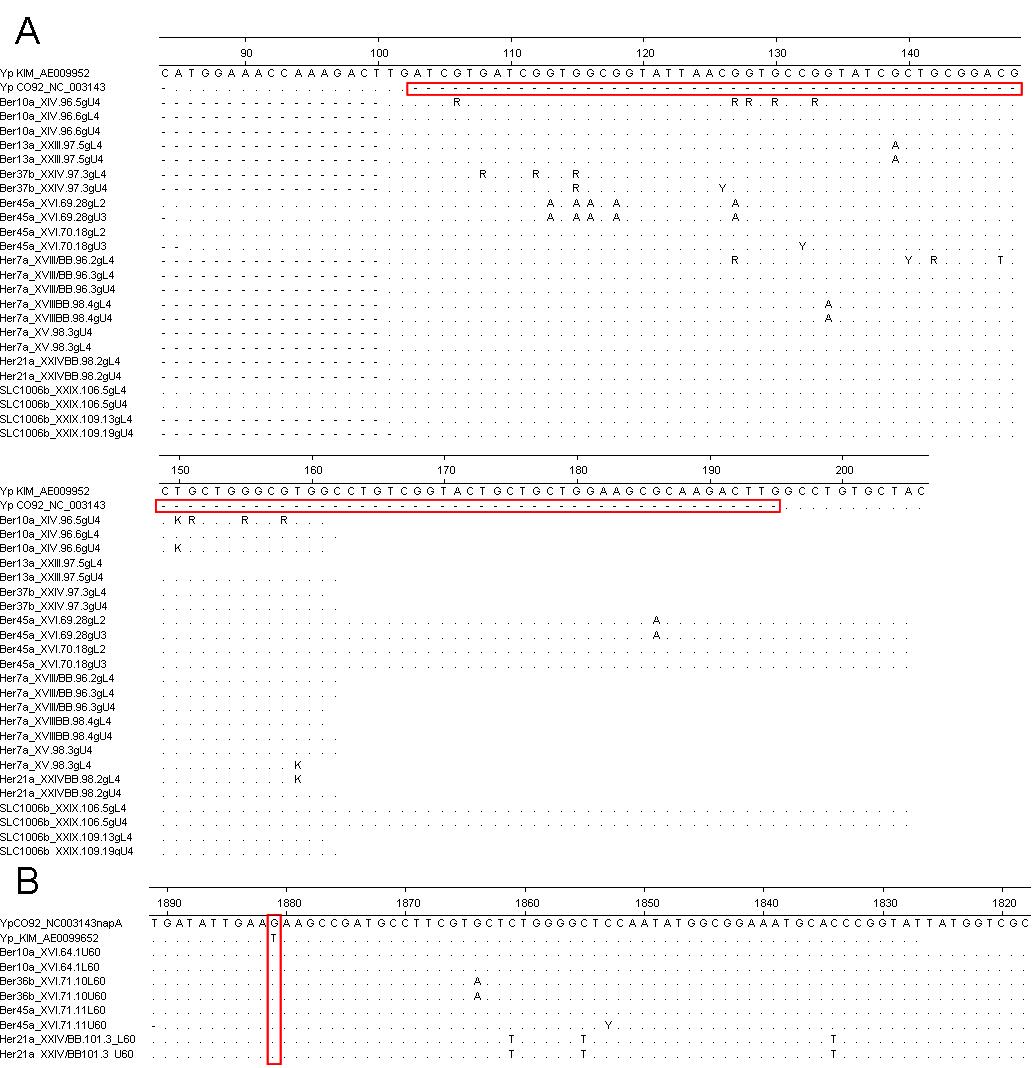
*

Figure S4: Alignment of the sequences encompassing five SNPs defining branch 0. The relevant codons are marked with red rectangles. A: s29 defining branch 0.PE2b (GCA), B: s31 defining branch 0.PE2a (GGA), C: s81excluding 0.PE4 and *Y. pseudotuberculosis* (AAA) and D-E: s82/s87 defining branch 0.PE (shortly before splitting into branches 1and 2).

**A**

**B**

**C**

**D**

**E**

**Figure S5: Alignment of the sequences encompassing five SNPs defining branch 1.** The relevant codons are marked with red rectangles. **A** and **B**: 1.ORI specific SNPs s2 (CGA) and s7 (ACC). **C-F**: branch 1 specific SNPs s11 (CAA), s12 (CGT), s13 (GCT) and s14 (ACG).

**A**

**B**

**C**

**D**

**E**

**F**

**Figure S6: Alignment of the sequences encompassing five SNPs defining branch 2.** The relevant codons are marked with red rectangles. **A**-**D**: branch 2 specific SNPs s15 (CAT), s17 (ACT), s18 (AAA) and s19 (CTA). **E**: s20 (GGT) defining 2.MED.


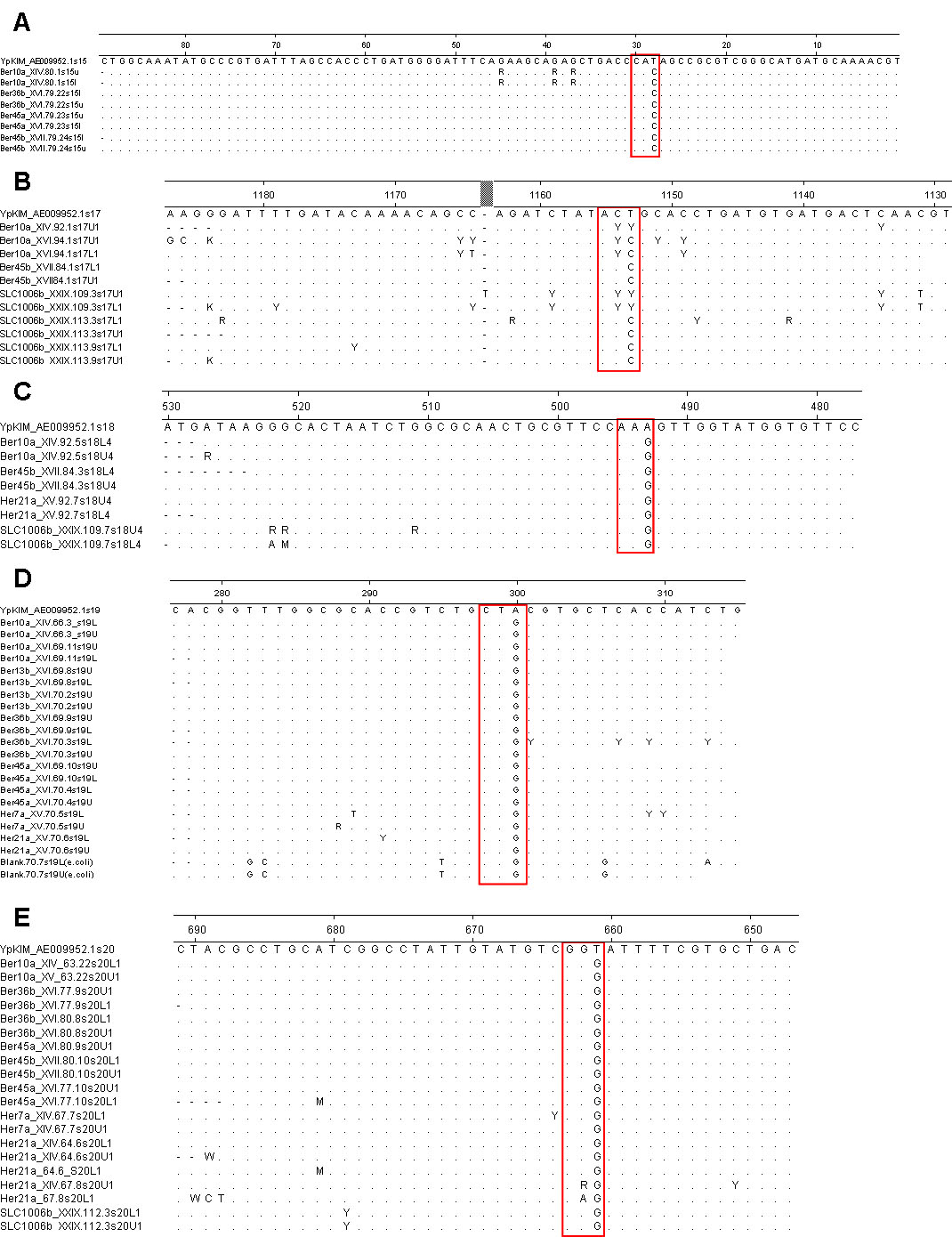

Supplement: Text S2 — Supplementary figures S1 to S6 of all aligned sequences (0.84 MB DOC) [file ppat.1001134.s002.doc]
