# Supplementary material for: Distinct Clones of Yersinia pestis Caused the Black Death
Source: PLoS Pathog. 2010 Oct 7;6(10):e1001134. doi: 10.1371/journal.ppat.1001134 (PMC2951374; doi:10.1371/journal.ppat.1001134)
Supplement: Table S1 — Results of the RDT analyses (0.10 MB DOC) [file ppat.1001134.s003.doc]

Table S1. Results of the RDT analysis. Code numbers for the samples, the results of the RDT for plague and the AgF1 concentrations are indicated. For the samples from Parma, the corresponding code used for genetic analysis is reported in parentheses.

| **Archaeological site** | **Code number** | **RDT bone** | **[AgF1] bone** |
| --- | --- | --- | --- |
| **Parma (Italy)** | **PR pit 1US 18** | **positive** | **>1.25-0.625 ng/ml** |
|  | **PR pit 1US 19 (PAR 119)** | **positive** | **>1.25-0.625 ng/ml** |
|  | PR pit 1US 20 (PAR 120) | negative | absent |
|  | **PR pit 2 US 202 ind. 01** | **positive** | **>0.625 ng/ml** |
|  | **PR pit 2 US 204 ind. 04** | **positive** | **>0.625 ng/ml** |
|  | PR pit 3 US 5 ind. 15 (PAR 315) | negative | absent |
|  | PR pit 3 US 19 ind.02 | negative | absent |
|  | **PR pit 3 US 20 ind. 11 (PAR 311)** | **positive** | **>0.625 ng/ml** |
|  | PR pit 3 US 32 ind.17 (PAR 317) | negative | absent |
|  | PR pit 3 US 53 ind. 5 | negative | absent |
|  | PR pit 3 US 55 ind. 9 | negative | absent |
|  | PR pit 3 US 64 ind. 08 (PAR 308) | negative | absent |
|  | PR pit 3 US 67 ind .06 | negative | absent |
|  | PR pit 3 US 68 ind. 13 | negative | absent |
|  | PR pit 3 US 69 ind. 07 | negative | absent |
|  | PR pit 3 US 78 ind. 10 | negative | absent |
|  | PR pit 3 US 93 ind. 03 (PAR 303) | negative | absent |
|  | **PR pit 3 US 97 ind. 16 (PAR 316)** | **positive** | **>0.625 ng/ml** |
|  | PR pit 3 US 99 ind. 14 | negative | absent |
| **Augsburg (Germany)** | **Man 3** | **positive** | **>0.625 ng/ml** |
|  | **Man 37** | **positive** | **>2.5-1.25 ng/ml** |
|  | **Man 9** | **positive** | **>1.25-0.625 ng/ml** |
|  | **Man 38** | **positive** | **>1.25-0.625 ng/ml** |
|  | Man 30 | negative | absent |
|  | Man 2 | negative | absent |
|  | Man 41 | negative | absent |
| **Bergen op Zoom**  **(The Netherlands)** | **BER 1** | **positive** | **>0.625 ng/ml** |
|  | BER 2 | negative | absent |
|  | **BER 3** | **positive** | **>0.625 ng/ml** |
|  | BER 6 | negative | absent |
|  | **BER 8** | **positive** | **>0.625 ng/ml** |
| **Hereford**  **(England)** | Her 7a | negative | absent |
|  | **Her 23a** | **positive** | **>0.625 ng/ml** |
|  | Her 24a | negative | absent |
|  | Her 27a | negative | absent |
|  | **Her 28a** | **positive** | **>1.25 ng/ml** |
|  | **Her 29a** | **positive** | **>1.25-0.625 ng/ml** |
|  | **Her 30a** | **positive** | **>1.25 ng/ml** |
| **Saint-Laurent-de-la-Cabrerisse (France)** | **SLC 1006** | **positive** | **>2.5-1.25 ng/ml** |
|  | **SLC 1010** | **positive** | **>0.625 ng/ml** |
|  | **SLC 1013** | **positive** | **>0.625 ng/ml** |
|  | **SLC 1014** | **positive** | **>0.625 ng/ml** |
|  | **SLC 1080** | **positive** | **>1.25-0.625 ng/ml** |
|  | SLC 1081 | negative | absent |
|  | SLC 1082 | negative | absent |
|  | **SLC 1083** | **positive** | **>1.25-0.625 ng/ml** |
|  | **SLC 1084** | **positive** | **>0.625 ng/ml** |
| **Negative controls** | Bös 844 | negative | absent |
|  | Bös 842 | negative | absent |
|  | SLC 128 | negative | absent |
|  | SLC 136 | negative | absent |
|  | SLC 156 | negative | absent |
|  | SLC 370 | negative | absent |
|  | SLC 144 | negative | absent |
|  | SLC 367 | negative | absent |
|  | BNK1 | negative | absent |
|  | BNK2 | negative | absent |
|  | BNK3 | negative | absent |
|  | BNK4 | negative | absent |
|  | BNK5 | negative | absent |
|  | BNK6 | negative | absent |
|  | BNK7 | negative | absent |
|  | BNK8 | negative | absent |
|  | BNK9 | negative | absent |
|  | BNK10 | negative | absent |
|  | BNK11 | negative | absent |
|  | BNK12 | negative | absent |
|  | BNK13 | negative | absent |
|  | BNK14 | negative | absent |
|  | BNK15 | negative | absent |
|  | BNK16 | negative | absent |
|  | BNK17 | negative | absent |
|  | BNK18 | negative | absent |
|  | BNK19 | negative | absent |
|  | BNK20 | negative | absent |
