# Supplementary material for: Distinct Clones of Yersinia pestis Caused the Black Death
Source: PLoS Pathog. 2010 Oct 7;6(10):e1001134. doi: 10.1371/journal.ppat.1001134 (PMC2951374; doi:10.1371/journal.ppat.1001134)
Supplement: Table S2 — Primers used in this study (0.09 MB DOC) [file ppat.1001134.s004.doc]

Table S2. Primers used in this study. Sequences are given in 5’🡪3’. PCR product length is given with and without primers (in parentheses). Note: primers for *glpD* by Drancourt *et al*. (2007; reference [14] in Text) are designed on *Y. pestis* Orientalis with the 93bp deletion, while primers gU3/gL2 and gU4/gL4 were designed on the *glp*D-gene of *Y. pestis* Medievalis lacking the deletion. *: 16S rDNA primers were used for amplification of the soil sample only.

| **Locus or branch** | **Primer pairs** | **Sequence 5’ - 3’** | **Annealing-temperature** | **Product length** | **Source** |
| --- | --- | --- | --- | --- | --- |
|  |  |  |  |  |  |
| *pla* | **YP12D** | cagcaggatatcaggaaaca | 55°C | 148bp (106bp) | Raoult  *et al*. 2000  (ref. [5] in Text) |
|  | **YP11R** | gcaagtccaatatatggcatag |  |  |  |
|  | **YP11D** | ctatgccatatattggacttgc |  |  |  |
|  | **YP10R** | gagccggatgtcttctcacg |  |  |  |
| *caf1* | **caf1 F1** | aaccagcccgcatcactctta | 56°C | 161bp (121bp) | *this study* |
|  | **caf1 R1** | atcacccgcggcatctgta |  |  |  |
|  | **U2** | aaataaccaccaattcactacaaaag | 55°C | 139bp (92bp) | *this study* |
|  | **L2** | tgagcgaacaaagaaatcctg |  |  |  |
| 16S rDNA* | **16S F3** | acacggtccagactcctacg | 58°C | 138bp (97bp) | *this study* |
|  | **16S R3** | aaactcaaccccttcctcctc |  |  |  |
| *rpoB* | **rpoB F1** | ttgacagcgataagggtaaaac | 55-56°C | 170bp (129bp) | *this study* |
|  | **rpoB R1** | caatgcacgcagaatgatg |  |  |  |
|  | **rpoB F2** | cgtaacggtagaggcgaagtg | 55-57°C | 164bp (120bp) | *this study* |
|  | **rpoB R2** | tgtggcatcagagtatcaaggtc |  |  |  |
| *glpD* | **glpD-F3** | cgctgtttcgaacattcaga | 54, 56, 58°C | 144 bp (104bp)  on 1.ORI | Drancourt  *et al.* 2007 |
|  | **glpD-R3** | ggccaaggcttcacttacca |  |  |  |
|  | **gU3** | tgtttcgaacattcagaggaaggtaa | 55°C | 167bp (122bp)  on 2.MED excluding deletion | *this study* |
|  | **gL2** | tttggaactggcggaagacg |  |  |  |
|  | **gU4** | ggaaggtaacatggaaaccaaagact | 59°C | 110bp (62bp)  on 2.MED excluding deletion | *this study* |
|  | **gL4** | cttccagcagcagtaccgacag |  |  |  |
| *napA* | **U60** | aggcggcggctggtcatac | 60-62°C | 113bp (74bp) | *this study* |
|  | **L60** | gagccgatggggtgctacga |  |  |  |
| 1.ORI | **s2 U2** | ggatggcaccttggaacagat | 55-62°C | 92bp (50bp) | *this study* |
|  | **s2 L2** | ctgcgcctaccaagactcgtt |  |  |  |
|  | **s7 U4** | aatctggagcgttggtttgagaag | 57°C | 130bp (86bp) | *this study* |
|  | **s7 L4** | aggcatgtgggttggcaatg |  |  |  |
| Branch 1 | **s11 U1** | atccgttccgccaactcttc | 56/62°C | 85bp (39bp) | *this study* |
|  | **s11 L1** | ccattaacaaactcaaccgtatctca |  |  |  |
|  | **s12 U1** | agagtccagcggtagctccagagt | 59-60°C | 82bp (36bp) | *this study* |
|  | **s12 L1** | ggaaaccccgattggtgatgac |  |  |  |
|  | **s13 U4** | ctggcctgttgcgtcatcagta | 59°C | 104bp (58bp) | *this study* |
|  | **s13 L4** | cgaaaatgggtgtaaaagcgaaat |  |  |  |
|  | **s14 U1** | ggtgaggtagtcgtcgtttgtg | 57°C | 100bp (56bp) | *this study* |
|  | **s14 L1** | ccgtaatgttcccttcttggatag |  |  |  |
| Branch 2 | **s15 U2** | tgcacgagcatcactttgtaa | 53°C | 130bp (89bp) | *this study* |
|  | **s15 L2** | acaggctaaagcagagcacc |  |  |  |
|  | **s17 U1** | aatacgcgcaaaattgttctga | 55°C | 102bp (59bp) | *this study* |
|  | **s17 U2** | acaagccgcaatcaatgagtc |  |  |  |
|  | **s18 U4** | aggtgtgggaatagctcaaaatgtt | 57°C | 103bp (54bp) | *this study* |
|  | **s18 L5** | ttacggttaatgggattgctgtta |  |  |  |
|  | **s19 L3** | atcttgtggcagattggcatc | 55°C | 80bp (39bp) | *this study* |
|  | **s19 U3** | ggatgtggatcgggactttc |  |  |  |
| 2.MED | **s20 L1** | ggcgcgtcacattgaatggtatt | 60°C | 92bp (49bp) | *this study* |
|  | **s20 U1** | ccgggaactcagagcacaga |  |  |  |
| 0.PE2.b | **s29U1** | ttcccttgatcaacggtcatacc | 57-58°C | 97bp (48bp) | *this study* |
|  | **s29L1** | ggtttatcgagtgggatcttcagttc |  |  |  |
| 0.PE2 | **s31U1** | gaagccgtacagatcgtgtttttc | 58°C | 118bp (70bp) | *this study* |
|  | **s31L1** | tgaggggtttgagctaggtgatag |  |  |  |
| Branch 0 | **s81U5** | gatcgtggtcagatggtcgc | U5/L1: 58°C U7/L1: 57-60°C | U5/L1: 89bp (48bp) | *this study* |
|  | **s81U7** | cgttgagcggtgtacggatt |  |  |  |
|  | **s81L1** | aagtgaaggagcgcactctgg |  | U7/L1: 117bp (75bp) |  |
| Branch 0 just before the split of branches  1 and 2 | **s82U1** | acagcttggggatgcttatcttc | 57°C | 87bp (42bp) | *this study* |
|  | **s82L1** | tgagtagcaggctttgcgagag |  |  |  |
|  | **s87U1** | tatgggtaaataccgcctgaat | 52-54°C | 93bp (48bp) | *this study* |
|  | **s87L1** | aagcgattgtatttgcctatcat |  |  |  |
