# Supplementary material for: Distinct Clones of Yersinia pestis Caused the Black Death
Source: PLoS Pathog. 2010 Oct 7;6(10):e1001134. doi: 10.1371/journal.ppat.1001134 (PMC2951374; doi:10.1371/journal.ppat.1001134)
Supplement: Table S3 — Amplified products obtained from the various samples analysed, using different sets of primers (0.86 MB DOC) [file ppat.1001134.s005.doc]

Table S3. Amplified products obtained from the various samples analysed, using different sets of primers. Amplification success rate is given as relative frequency based on the number of attempts. Samples from the same locality are listed together. (BK) indicates negative controls.

| **Sample** | | **Extraction** | **YP12D/YP11R** | **YP11D/YP10R** | **caf1 F1/R1** | **caf1 U2/L2** | **rpoB1** | **rpoB2** | **aspA** | **napA 613** | **glpD gU4/gL4** | **glpD gU3/gL2** | **glpD-F3/R3** | **s2** | **s7** | **s11** | **s12** | **s13** | **s14** | **s15** | **s17** | **s18** | **s19** | **s20** | **s29** | **s31** | **s81** | **s82** | **s87** |
| --- | --- | --- | --- | --- | --- | --- | --- | --- | --- | --- | --- | --- | --- | --- | --- | --- | --- | --- | --- | --- | --- | --- | --- | --- | --- | --- | --- | --- | --- |
| Ber1 | A  Tooth | XXIBB |  | 0/1 |  |  |  |  |  |  |  |  |  |  |  |  |  |  |  |  |  |  |  |  |  |  |  |  |  |
|  | B  Tooth | XII | 0/2 |  |  |  |  |  |  |  |  |  |  |  |  |  |  |  |  |  |  |  |  |  |  |  |  |  |  |
|  |  | XIV |  | 0/3 |  |  |  |  |  |  |  |  |  |  |  |  |  |  |  |  |  |  |  |  |  |  |  |  |  |
| Ber2 | B  Tooth | XII | 0/2 |  |  |  |  |  |  |  |  |  |  |  |  |  |  |  |  |  |  |  |  |  |  |  |  |  |  |
|  |  | XIV |  | 0/3 |  |  |  |  |  |  |  |  |  |  |  |  |  |  |  |  |  |  |  |  |  |  |  |  |  |
| Ber3 | A  Tooth | XXBB |  | 1/1 | 0/1 | 0/1 | 0/1 | 0/1 |  |  |  | 0/2 | 0/2 |  |  |  |  |  |  |  |  |  |  |  |  |  |  |  |  |
|  |  | XIII |  | 0/1 |  |  |  |  |  |  |  |  |  |  |  |  |  |  |  |  |  |  |  |  |  |  |  |  |  |
|  |  | XV | 0/1 |  |  |  |  |  |  |  |  |  |  |  |  |  |  |  |  |  |  |  |  |  |  |  |  |  |  |
|  | B  Tooth | XII | 0/2 |  |  |  |  |  |  |  |  |  |  |  |  |  |  |  |  |  |  |  |  |  |  |  |  |  |  |
|  | C  Tooth | XVI |  | 0/1 |  |  |  |  |  |  |  |  |  |  |  |  |  |  |  |  |  |  |  |  |  |  |  |  |  |
| Ber4 | A  Tooth | XXIBB |  | 0/1 |  |  |  |  |  |  |  |  |  |  |  |  |  |  |  |  |  |  |  |  |  |  |  |  |  |
|  | B  Tooth | XII | 0/2 |  |  |  |  |  |  |  |  |  |  |  |  |  |  |  |  |  |  |  |  |  |  |  |  |  |  |
|  |  | XIV |  | 0/3 |  |  |  |  |  |  |  |  |  |  |  |  |  |  |  |  |  |  |  |  |  |  |  |  |  |
| Ber5 | A  Tooth | XXIBB |  | 0/1 |  |  |  |  |  |  |  |  |  |  |  |  |  |  |  |  |  |  |  |  |  |  |  |  |  |
|  | B  Tooth | XII |  | 0/1 |  |  |  |  |  |  |  |  |  |  |  |  |  |  |  |  |  |  |  |  |  |  |  |  |  |
|  |  | XIV |  | 0/3 |  |  |  |  |  |  |  |  |  |  |  |  |  |  |  |  |  |  |  |  |  |  |  |  |  |
| Ber6 | A  Tooth | XXIBB |  | 0/1 |  |  |  |  |  |  |  |  |  |  |  |  |  |  |  |  |  |  |  |  |  |  |  |  |  |
|  | B  Tooth | XII | 0/1 |  |  |  |  |  |  |  |  |  |  |  |  |  |  |  |  |  |  |  |  |  |  |  |  |  |  |
|  |  | XIV |  | 0/3 |  |  |  |  |  |  |  |  |  |  |  |  |  |  |  |  |  |  |  |  |  |  |  |  |  |
| Ber7 | A  Tooth | XXBB |  | 0/1 |  |  |  |  |  |  |  |  |  |  |  |  |  |  |  |  |  |  |  |  |  |  |  |  |  |
|  | B  Tooth | XIII |  | 0/1 |  |  |  |  |  |  |  |  |  |  |  |  |  |  |  |  |  |  |  |  |  |  |  |  |  |
|  |  | XIV |  | 0/3 |  |  |  |  |  |  |  |  |  |  |  |  |  |  |  |  |  |  |  |  |  |  |  |  |  |
| Ber8 | A  Tooth | XXBB |  | 0/1 |  |  |  |  |  |  |  |  |  |  |  |  |  |  |  |  |  |  |  |  |  |  |  |  |  |
|  | B  Tooth | XII | 0/2 |  |  |  |  |  |  |  |  |  |  |  |  |  |  |  |  |  |  |  |  |  |  |  |  |  |  |
|  |  | XIV |  | 0/3 |  |  |  |  |  |  |  |  |  |  |  |  |  |  |  |  |  |  |  |  |  |  |  |  |  |
| Ber9 | A  Tooth | XXBB |  | 1/1 | 0/1 | 0/1 | 0/1 | 0/1 |  |  |  | 0/2 | 0/2 |  |  |  |  |  |  |  |  |  |  |  |  |  |  |  |  |
|  |  | XV | 0/1 |  |  |  |  |  |  |  |  |  |  |  |  |  |  |  |  |  |  |  |  |  |  |  |  |  |  |
|  | B  Tooth | XII | 0/2 |  |  |  |  |  |  |  |  |  |  |  |  |  |  |  |  |  |  |  |  |  |  |  |  |  |  |
|  |  | XIV |  | 0/3 |  |  |  |  |  |  |  |  |  |  |  |  |  |  |  |  |  |  |  |  |  |  |  |  |  |
|  | C  Tooth | XVI |  | 0/1 |  |  |  |  |  |  |  |  |  |  |  |  |  |  |  |  |  |  |  |  |  |  |  |  |  |
| Ber10 | A  Tooth | XXBB |  | 1/1 | 1/1 | 1/1 | 1/1 | 1/1 |  |  |  | 0/2 | 0/2 |  |  |  |  |  |  |  |  |  |  |  |  |  |  |  |  |
|  |  | XIV | 1/1 | 3/3 |  |  | 0/1 |  | 2/2 | 1/2 | 2/3 | 1/2 | 0/2 | 1/1 |  |  |  |  | 1/1 | 1/1 |  |  | 1/1 | 1/1 |  |  | 0/1 |  |  |
|  |  | XVI |  | 1/1 | 1/1 |  |  |  |  |  |  | 1/1 |  | 1/1 |  | 1/1 | 1/1 | 1/1 | 1/1 | 0/1 | 1/1 | 1/1 | 1/1 |  | 1/1 | 1/1 | 1/1 | 1/1 | 1/1 |
|  | B  Tooth | XII | 0/2 |  |  |  |  |  |  |  |  |  |  |  |  |  |  |  |  |  |  |  |  |  |  |  |  |  |  |
|  |  | XIV |  | 0/3 |  |  |  |  |  |  |  |  |  |  |  |  |  |  |  |  |  |  |  |  |  |  |  |  |  |
|  | C  Tooth | XVI |  | 0/1 |  |  |  |  |  |  |  |  |  |  |  |  |  |  |  |  |  |  |  |  |  |  |  |  |  |
| Ber11 | A  Tooth | XVI |  | 0/1 |  |  |  |  |  |  |  |  |  |  |  |  |  |  |  |  |  |  |  |  |  |  |  |  |  |
|  | B  Tooth | XXII |  | 0/1 |  |  |  |  |  |  |  |  |  |  |  |  |  |  |  |  |  |  |  |  |  |  |  |  |  |
| Ber12 | A  Tooth | XVI |  | 1/1 | 0/1 |  |  |  |  | 0/1 | 0/1 | 0/2 |  | 1/2 |  |  |  |  | 1/2 |  |  |  | 1/2 |  |  |  |  |  |  |
| Ber13 | A  Tooth | XXIII |  | 1/1 |  |  |  |  |  |  |  |  |  |  |  |  |  |  |  |  |  |  |  |  |  |  |  |  |  |
|  | B  Tooth | XVI |  | 1/1 | 0/1 |  |  |  |  | 0/1 | 1/1 | 0/2 |  | 1/2 |  | 0/1 | 1/1 |  | 1/2 | 0/1 |  |  | 2/2 |  | 0/1 | 1/1 | 1/3 | 0/1 |  |
| Ber14 | A  Tooth | XVI |  | 0/1 |  |  |  |  |  |  |  |  |  |  |  |  |  |  |  |  |  |  |  |  |  |  |  |  |  |
|  |  | XXIV |  | 0/1 |  |  |  |  |  |  |  |  |  |  |  |  |  |  |  |  |  |  |  |  |  |  |  |  |  |
| Ber15 | A  Tooth | XVII | 0/1 |  |  |  |  |  |  |  |  |  |  |  |  |  |  |  |  |  |  |  |  |  |  |  |  |  |  |
|  | B  Tooth | XXIII |  | 0/1 |  |  |  |  |  |  |  |  |  |  |  |  |  |  |  |  |  |  |  |  |  |  |  |  |  |
| Ber16 | A  Tooth | XVII | 1/1 |  |  |  |  |  |  |  | 0/1 |  |  |  |  | 0/1 | 0/1 |  |  | 0/1 |  |  |  |  |  |  | 1/1 |  |  |
|  | B  Tooth | XXIV |  | 0/1 |  |  |  |  |  |  |  |  |  |  |  |  |  |  |  |  |  |  |  |  |  |  |  |  |  |
| Ber17 | A  Tooth | XVII | 0/1 |  |  |  |  |  |  |  |  |  |  |  |  |  |  |  |  |  |  |  |  |  |  |  |  |  |  |
| Ber18 | A  Tooth | XVIII | 0/1 |  |  |  |  |  |  |  |  |  |  |  |  |  |  |  |  |  |  |  |  |  |  |  |  |  |  |
| Ber19 | A  Tooth | XVII | 0/1 |  |  |  |  |  |  |  |  |  |  |  |  |  |  |  |  |  |  |  |  |  |  |  |  |  |  |
| Ber20 | A  Tooth | XVII | 0/1 |  |  |  |  |  |  |  |  |  |  |  |  |  |  |  |  |  |  |  |  |  |  |  |  |  |  |
|  | B  Tooth | XXII |  | 0/1 |  |  |  |  |  |  |  |  |  |  |  |  |  |  |  |  |  |  |  |  |  |  |  |  |  |
| Ber21 | A  Tooth | XXIV | 0/1 |  |  |  |  |  |  |  |  |  |  |  |  |  |  |  |  |  |  |  |  |  |  |  |  |  |  |
| Ber22 | A  Tooth | XVII | 0/1 |  |  |  |  |  |  |  |  |  |  |  |  |  |  |  |  |  |  |  |  |  |  |  |  |  |  |
| Ber24 | B  Tooth | XVIII | 0/1 |  |  |  |  |  |  |  |  |  |  |  |  |  |  |  |  |  |  |  |  |  |  |  |  |  |  |
| Ber25 | A  Tooth | XXIV |  | 0/1 |  |  |  |  |  |  |  |  |  |  |  |  |  |  |  |  |  |  |  |  |  |  |  |  |  |
| Ber26 | A  Tooth | XVI |  | 0/1 |  |  |  |  |  |  |  |  |  |  |  |  |  |  |  |  |  |  |  |  |  |  |  |  |  |
|  | B  Tooth | XVII | 0/1 |  |  |  |  |  |  |  |  |  |  |  |  |  |  |  |  |  |  |  |  |  |  |  |  |  |  |
| Ber27 | A  Tooth | XXIV |  | 0/1 |  |  |  |  |  |  |  |  |  |  |  |  |  |  |  |  |  |  |  |  |  |  |  |  |  |
| Ber28 | A  Tooth | XXIV |  | 0/1 |  |  |  |  |  |  |  |  |  |  |  |  |  |  |  |  |  |  |  |  |  |  |  |  |  |
| Ber29 | B  Tooth | XXIII |  | 0/1 |  |  |  |  |  |  |  |  |  |  |  |  |  |  |  |  |  |  |  |  |  |  |  |  |  |
| Ber30 | B  Tooth | XVIII | 0/1 |  |  |  |  |  |  |  |  |  |  |  |  |  |  |  |  |  |  |  |  |  |  |  |  |  |  |
| Ber31 | A  Tooth | XVIII | 0/1 |  |  |  |  |  |  |  |  |  |  |  |  |  |  |  |  |  |  |  |  |  |  |  |  |  |  |
| Ber32 | A  Tooth | XXIII |  | 0/1 |  |  |  |  |  |  |  |  |  |  |  |  |  |  |  |  |  |  |  |  |  |  |  |  |  |
|  | B  Tooth | XXIV |  | 0/1 |  |  |  |  |  |  |  |  |  |  |  |  |  |  |  |  |  |  |  |  |  |  |  |  |  |
| Ber33 | A  Tooth | XVII | 0/1 |  |  |  |  |  |  |  |  |  |  |  |  |  |  |  |  |  |  |  |  |  |  |  |  |  |  |
| Ber34 | A  Tooth | XXIII |  | 0/1 |  |  |  |  |  |  |  |  |  |  |  |  |  |  |  |  |  |  |  |  |  |  |  |  |  |
|  | B  Tooth | XVI |  | 0/1 |  |  |  |  |  |  |  |  |  |  |  |  |  |  |  |  |  |  |  |  |  |  |  |  |  |
| Ber35 | A  Tooth | XXIII |  | 0/1 |  |  |  |  |  |  |  |  |  |  |  |  |  |  |  |  |  |  |  |  |  |  |  |  |  |
|  | B  Tooth | XVIII | 0/1 |  |  |  |  |  |  |  |  |  |  |  |  |  |  |  |  |  |  |  |  |  |  |  |  |  |  |
| Ber36 | A  Tooth | XXIII |  | 0/1 |  |  |  |  |  |  |  |  |  |  |  |  |  |  |  |  |  |  |  |  |  |  |  |  |  |
|  | B  Tooth | XVI |  | 1/1 | 1/1 |  |  |  |  | 1/1 | 0/1 | 0/2 | 0/1 | 2/2 |  | 1/1 | 1/2 |  | 2/2 | 1/1 |  |  | 2/2 | 3/3 | 1/1 | 1/1 | 1/3 | 1/1 |  |
| Ber37 | B  Tooth | XXIV |  | 1/1 |  |  |  |  |  |  | 1/1 |  |  |  |  |  | 1/1 |  |  |  |  |  |  |  |  |  |  |  |  |
|  | A  Tooth | XXV |  | 1/1 |  |  |  |  |  |  |  |  |  |  |  |  |  |  |  |  |  |  |  |  |  |  |  |  |  |
| Ber38 | A  Tooth | XXII |  | 0/1 |  |  |  |  |  |  |  |  |  |  |  |  |  |  |  |  |  |  |  |  |  |  |  |  |  |
| Ber39 | B  Tooth | XVIII | 0/1 |  |  |  |  |  |  |  |  |  |  |  |  |  |  |  |  |  |  |  |  |  |  |  |  |  |  |
| Ber40 | B  Tooth | XVII | 0/1 |  |  |  |  |  |  |  |  |  |  |  |  |  |  |  |  |  |  |  |  |  |  |  |  |  |  |
| Ber41 | B  Tooth | XVI |  | 0/1 |  |  |  |  |  |  |  |  |  |  |  |  |  |  |  |  |  |  |  |  |  |  |  |  |  |
| Ber42 | A  Tooth | XVII | 0/1 |  |  |  |  |  |  |  |  |  |  |  |  |  |  |  |  |  |  |  |  |  |  |  |  |  |  |
| Ber43 | A  Tooth | XXV |  | 0/1 |  |  |  |  |  |  |  |  |  |  |  |  |  |  |  |  |  |  |  |  |  |  |  |  |  |
| Ber45 | A  Tooth | XVI |  | 1/1 | 1/1 |  | 1/1 |  | 1/1 |  |  | 2/2 |  | 2/2 |  | 1/1 | 2/2 |  | 2/2 | 1/1 |  |  | 2/2 | 2/2 | 1/1 | 1/1 | 3/3 | 1/1 |  |
|  | B  Tooth | XVII | 1/1 |  |  |  |  |  |  |  |  |  |  |  |  | 1/1 | 1/1 | 1/1 |  | 1/1 | 1/1 | 1/1 |  | 1/1 |  |  | 1/1 |  | 1/1 |
| BNK1  (BK) | A  Tooth | XLI |  | 0/2 |  |  |  |  |  |  |  |  |  |  |  |  | 0/1 |  |  |  |  |  | 0/1 |  |  |  |  |  |  |
|  | B  Tooth | XLII |  | 0/2 |  |  |  |  |  |  |  |  |  |  |  |  | 0/1 |  |  |  |  |  | 0/1 |  |  |  |  |  |  |
| BNK2  (BK) | A  Tooth | XLI |  | 0/3 |  |  |  |  |  |  |  |  |  |  |  |  | 0/1 |  |  |  |  |  | 0/1 |  |  |  |  |  |  |
|  | B  Tooth | XLII |  | 0/2 |  |  |  |  |  |  |  |  |  |  |  |  | 0/1 |  |  |  |  |  | 0/1 |  |  |  |  |  |  |
| BNK3  (BK) | A  Tooth | XLI |  | 0/2 |  |  |  |  |  |  |  |  |  |  |  |  | 0/1 |  |  |  |  |  | 0/1 |  |  |  |  |  |  |
|  | B  Tooth | XLII |  | 0/2 |  |  |  |  |  |  |  |  |  |  |  |  | 0/1 |  |  |  |  |  | 0/1 |  |  |  |  |  |  |
| BNK4  (BK) | A  Tooth | XLI |  | 0/2 |  |  |  |  |  |  |  |  |  |  |  |  | 0/1 |  |  |  |  |  | 0/1 |  |  |  |  |  |  |
|  | B  Tooth | XLII |  | 0/2 |  |  |  |  |  |  |  |  |  |  |  |  | 0/1 |  |  |  |  |  | 0/1 |  |  |  |  |  |  |
| BNK5  (BK) | A  Tooth | XLI |  | 0/2 |  |  |  |  |  |  |  |  |  |  |  |  | 0/1 |  |  |  |  |  | 0/1 |  |  |  |  |  |  |
|  | B  Tooth | XLII |  | 0/2 |  |  |  |  |  |  |  |  |  |  |  |  | 0/1 |  |  |  |  |  | 0/1 |  |  |  |  |  |  |
| BNK6  (BK) | A  Tooth | XLI |  | 0/2 |  |  |  |  |  |  |  |  |  |  |  |  | 0/1 |  |  |  |  |  | 0/1 |  |  |  |  |  |  |
|  | B  Tooth | XLII |  | 0/2 |  |  |  |  |  |  |  |  |  |  |  |  | 0/1 |  |  |  |  |  | 0/1 |  |  |  |  |  |  |
| BNK7  (BK) | A  Tooth | XLI |  | 0/2 |  |  |  |  |  |  |  |  |  |  |  |  | 0/1 |  |  |  |  |  | 0/1 |  |  |  |  |  |  |
|  | B  Tooth | XLII |  | 0/2 |  |  |  |  |  |  |  |  |  |  |  |  | 0/1 |  |  |  |  |  | 0/1 |  |  |  |  |  |  |
| BNK8  (BK) | A  Tooth | XLI |  | 0/2 |  |  |  |  |  |  |  |  |  |  |  |  | 0/1 |  |  |  |  |  | 0/1 |  |  |  |  |  |  |
|  | B  Tooth | XLII |  | 0/2 |  |  |  |  |  |  |  |  |  |  |  |  | 0/1 |  |  |  |  |  | 0/1 |  |  |  |  |  |  |
| BNK9  (BK) | A  Tooth | XLI |  | 0/2 |  | 0/1 |  |  |  |  | 0/1 |  |  |  |  |  | 0/1 |  |  |  |  |  | 0/2 |  |  |  |  |  |  |
|  | B  Tooth | XLII |  | 0/2 |  |  |  |  |  |  |  |  |  |  |  |  | 0/1 |  |  |  |  |  | 0/1 |  |  |  |  |  |  |
| BNK10  (BK) | A  Tooth | XLI |  | 0/2 |  | 0/1 |  |  |  |  | 0/1 |  |  |  |  |  | 0/1 |  |  |  |  |  | 0/2 |  |  |  |  |  |  |
|  | B  Tooth | XLII |  | 0/3 |  |  |  |  |  |  |  |  |  |  |  |  | 0/1 |  |  |  |  |  | 0/1 |  |  |  |  |  |  |
| BNK11  (BK) | A  Tooth | XLI |  | 0/2 |  |  |  |  |  |  |  |  |  |  |  |  | 0/1 |  |  |  |  |  | 0/1 |  |  |  |  |  |  |
|  | B  Tooth | XLII |  | 0/2 |  |  |  |  |  |  |  |  |  |  |  |  | 0/1 |  |  |  |  |  | 0/1 |  |  |  |  |  |  |
| BNK12  (BK) | A  Tooth | XLI |  | 0/2 |  |  |  |  |  |  |  |  |  |  |  |  | 0/1 |  |  |  |  |  | 0/1 |  |  |  |  |  |  |
|  | B  Tooth | XLII |  | 0/2 |  |  |  |  |  |  |  |  |  |  |  |  | 0/1 |  |  |  |  |  | 0/1 |  |  |  |  |  |  |
| BNK13  (BK) | A  Tooth | XLI |  | 0/2 |  |  |  |  |  |  |  |  |  |  |  |  | 0/1 |  |  |  |  |  | 0/1 |  |  |  |  |  |  |
|  | B  Tooth | XLII |  | 0/2 |  |  |  |  |  |  |  |  |  |  |  |  | 0/1 |  |  |  |  |  | 0/1 |  |  |  |  |  |  |
| BNK14  (BK) | A  Tooth | XLI |  | 0/2 |  |  |  |  |  |  |  |  |  |  |  |  | 0/1 |  |  |  |  |  | 0/1 |  |  |  |  |  |  |
|  | B  Tooth | XLII |  | 0/2 |  |  |  |  |  |  |  |  |  |  |  |  | 0/1 |  |  |  |  |  | 0/1 |  |  |  |  |  |  |
| BNK15  (BK) | A  Tooth | XLI |  | 0/2 |  |  |  |  |  |  |  |  |  |  |  |  | 0/1 |  |  |  |  |  | 0/1 |  |  |  |  |  |  |
|  | B  Tooth | XLII |  | 0/2 |  |  |  |  |  |  |  |  |  |  |  |  | 0/1 |  |  |  |  |  | 0/1 |  |  |  |  |  |  |
| BNK16  (BK) | A  Tooth | XLI |  | 0/2 |  |  |  |  |  |  |  |  |  |  |  |  | 0/1 |  |  |  |  |  | 0/1 |  |  |  |  |  |  |
|  | B  Tooth | XLII |  | 0/2 |  |  |  |  |  |  |  |  |  |  |  |  | 0/1 |  |  |  |  |  | 0/1 |  |  |  |  |  |  |
| BNK17  (BK) | A  Tooth | XLII |  | 0/2 |  |  |  |  |  |  |  |  |  |  |  |  | 0/1 |  |  |  |  |  | 0/1 |  |  |  |  |  |  |
|  | B  Tooth | XLII |  | 0/2 |  |  |  |  |  |  |  |  |  |  |  |  | 0/1 |  |  |  |  |  | 0/1 |  |  |  |  |  |  |
| BNK18  (BK) | A  Tooth | XLII |  | 0/2 |  |  |  |  |  |  |  |  |  |  |  |  | 0/1 |  |  |  |  |  | 0/1 |  |  |  |  |  |  |
|  | B  Tooth | XLII |  | 0/2 |  |  |  |  |  |  |  |  |  |  |  |  | 0/1 |  |  |  |  |  | 0/1 |  |  |  |  |  |  |
| BNK19  (BK) | A  Tooth | XLII | 0/1 | 0/2 |  |  |  |  |  |  |  |  |  |  |  |  | 0/1 |  |  |  |  |  | 0/1 |  |  |  |  |  |  |
|  | B  Tooth | XLII |  | 0/2 |  |  |  |  |  |  |  |  |  |  |  |  | 0/1 |  |  |  |  |  | 0/1 |  |  |  |  |  |  |
| BNK20  (BK) | A  Tooth | XLII |  | 0/2 |  |  |  |  |  |  |  |  |  |  |  |  | 0/1 |  |  |  |  |  | 0/1 |  |  |  |  |  |  |
|  | B  Tooth | XLII |  | 0/2 |  |  |  |  |  |  |  |  |  |  |  |  | 0/1 |  |  |  |  |  | 0/1 |  |  |  |  |  |  |
| Her2 | A  Tooth | XVIIBB | 0/1 | 0/1 |  |  | 0/1 |  |  |  |  |  |  |  |  |  |  |  |  |  |  |  |  |  |  |  |  |  |  |
| Her5 | A  Tooth | XVIIBB | 0/1 | 0/1 |  |  | 0/1 |  |  |  |  |  |  |  |  |  |  |  |  |  |  |  |  |  |  |  |  |  |  |
| Her7 | A  Tooth | XVIIIBB | 1/1 | 1/1 |  |  | 0/1 |  |  | 0/2 | 4/4 |  |  |  |  |  |  |  |  |  |  |  |  |  |  |  |  |  |  |
|  |  | XV | 1/1 |  |  | 0/1 | 0/1 | 0/1 |  | 0/3 | 1/1 | 0/3 |  | 2/2 |  | 1/1 | 2/2 | 1/1 | 1/2 | 0/1 | 0/1 | 0/1 | 2/2 | 1/2 | 1/1 | 1/1 | 0/3 | 1/1 | 0/1 |
|  |  | XXI | 1/2 | 2/3 |  | 0/2 |  |  |  |  |  |  |  |  |  |  |  |  |  |  |  |  |  |  |  |  |  |  |  |
| Her8 | A  Tooth | XVIIIBB | 0/1 | 0/1 |  |  | 0/1 |  |  |  |  |  |  |  |  |  |  |  |  |  |  |  |  |  |  |  |  |  |  |
| Her17 | A  Tooth | XVIIBB | 0/1 | 0/1 |  |  | 0/1 |  |  |  |  |  |  |  |  |  |  |  |  |  |  |  |  |  |  |  |  |  |  |
| Her18 | A  Tooth | XVIIBB | 0/1 | 0/1 |  |  | 0/1 |  |  |  |  |  |  |  |  |  |  |  |  |  |  |  |  |  |  |  |  |  |  |
| Her21 | A  Tooth | XXIVBB | 1/1 | 1/1 |  |  | 0/1 |  |  | 1/2 | 1/1 |  |  |  |  |  |  |  |  |  |  |  |  |  |  |  |  |  |  |
|  |  | XV | 1/1 | 1/1 |  | 1/3 | 0/1 | 0/1 | 1/1 | 0/3 | 0/2 | 0/3 | 0/1 | 2/2 |  | 1/1 | 1/2 | 1/1 | 2/2 | 0/1 | 0/2 | 1/1 | 2/2 | 2/2 | 1/1 | 1/1 | 0/3 | 1/1 | 1/1 |
|  | B  Tooth | XXI | 0/1 | 0/4 |  | 0/2 |  |  |  |  |  |  |  |  |  |  |  |  |  |  |  |  |  |  |  |  |  |  |  |
| Her22 | A  Tooth | XXIVBB | 0/1 | 0/1 |  |  | 0/1 |  |  |  |  |  |  |  |  |  |  |  |  |  |  |  |  |  |  |  |  |  |  |
|  |  | XXI | 0/1 | 0/4 |  |  |  |  |  |  |  |  |  |  |  |  |  |  |  |  |  |  |  |  |  |  |  |  |  |
| Her23 | A  Tooth | XXIVBB | 0/1 | 0/1 |  |  | 0/1 |  |  |  |  |  |  |  |  |  |  |  |  |  |  |  |  |  |  |  |  |  |  |
| Her24 | A  Tooth | XXIVBB | 0/1 | 0/1 |  |  | 0/1 |  |  |  |  |  |  |  |  |  |  |  |  |  |  |  |  |  |  |  |  |  |  |
| Her25 | A  Tooth | XXIVBB | 0/1 | 0/1 |  |  | 0/1 |  |  |  |  |  |  |  |  |  |  |  |  |  |  |  |  |  |  |  |  |  |  |
|  |  | XXI | 0/1 | 0/4 |  |  |  |  |  |  |  |  |  |  |  |  |  |  |  |  |  |  |  |  |  |  |  |  |  |
| Her26 | A  Tooth | XXIVBB | 0/1 | 0/1 |  |  | 0/1 |  |  |  |  |  |  |  |  |  |  |  |  |  |  |  |  |  |  |  |  |  |  |
|  |  | XXI | 0/1 | 0/4 |  |  |  |  |  |  |  |  |  |  |  |  |  |  |  |  |  |  |  |  |  |  |  |  |  |
| SLC  1006 | A  Tooth | XXVIII |  | 0/1 |  |  |  |  |  |  |  |  |  |  |  |  |  |  |  |  |  |  |  |  |  |  |  |  |  |
|  | B  Tooth | XXIX |  | 2/2 |  | 1/1 |  |  |  | 0/4 | 2/2 |  |  | 1/4 | 1/2 | 2/2 | 2/2 | 2/2 | 1/1 |  | 2/2 | 1/1 |  | 1/2 |  |  |  | 1/1 |  |
| SLC  1010 | A  Tooth | XXVIII |  | 0/1 |  |  |  |  |  |  |  |  |  |  |  |  |  |  |  |  |  |  |  |  |  |  |  |  |  |
|  | B  Tooth | XXIX |  | 0/1 |  |  |  |  |  |  |  |  |  |  |  |  |  |  |  |  |  |  |  |  |  |  |  |  |  |
| SLC  1013 | A  Tooth | XXVIII |  | 0/1 |  |  |  |  |  |  |  |  |  |  |  |  |  |  |  |  |  |  |  |  |  |  |  |  |  |
|  | B  Tooth | XXIX |  | 0/1 |  |  |  |  |  |  |  |  |  |  |  |  |  |  |  |  |  |  |  |  |  |  |  |  |  |
| SLC  1080 | A  Tooth | XXVIII |  | 0/1 |  |  |  |  |  |  |  |  |  |  |  |  |  |  |  |  |  |  |  |  |  |  |  |  |  |
| SLC  1081 | A  Tooth | XXXI |  | 0/1 |  |  |  |  |  |  |  |  |  |  |  |  |  |  |  |  |  |  |  |  |  |  |  |  |  |
|  | B  Tooth | XXIX |  | 0/1 |  |  |  |  |  |  |  |  |  |  |  |  |  |  |  |  |  |  |  |  |  |  |  |  |  |
| SLC  1083 | A  Tooth | XXIX |  | 0/1 |  |  |  |  |  |  |  |  |  |  |  |  |  |  |  |  |  |  |  |  |  |  |  |  |  |
| SLC  128  (BK) | A  Tooth | XXXI |  | 0/1 |  |  |  |  |  |  |  |  |  |  |  |  |  |  |  |  |  |  |  |  |  |  |  |  |  |
|  | B  Tooth | XXIX |  | 0/1 |  |  |  |  |  |  |  |  |  |  |  |  |  |  |  |  |  |  |  |  |  |  |  |  |  |
| SLC  136  (BK) | A  Tooth | XXVIII |  | 0/1 |  |  |  |  |  |  |  |  |  |  |  |  |  |  |  |  |  |  |  |  |  |  |  |  |  |
|  | B  Tooth | XXIX |  | 0/1 |  |  |  |  |  |  |  |  |  |  |  |  |  |  |  |  |  |  |  |  |  |  |  |  |  |
| SLC  156  (BK) | A  Tooth | XXVIII |  | 0/1 |  |  |  |  |  |  |  |  |  |  |  |  |  |  |  |  |  |  |  |  |  |  |  |  |  |
|  | B  Tooth | XXIX |  | 0/1 |  |  |  |  |  |  |  |  |  |  |  |  |  |  |  |  |  |  |  |  |  |  |  |  |  |
| SCL  370  (BK) | A  Tooth | XXVIII |  | 0/1 |  |  |  |  |  |  |  |  |  |  |  |  |  |  |  |  |  |  |  |  |  |  |  |  |  |
|  | B  Tooth | XXIX |  | 0/1 |  |  |  |  |  |  |  |  |  |  |  |  |  |  |  |  |  |  |  |  |  |  |  |  |  |
| SLC  144  (BK) | A  Tooth | XXXI |  | 0/1 |  |  |  |  |  |  |  |  |  |  |  |  |  |  |  |  |  |  |  |  |  |  |  |  |  |
|  | B  Tooth | XXIX |  | 0/1 |  |  |  |  |  |  |  |  |  |  |  |  |  |  |  |  |  |  |  |  |  |  |  |  |  |
| SLC  367  (BK) | A  Tooth | XXVIII |  | 0/1 |  |  |  |  |  |  |  |  |  |  |  |  |  |  |  |  |  |  |  |  |  |  |  |  |  |
|  | B  Tooth | XXIX |  | 0/1 |  |  |  |  |  |  |  |  |  |  |  |  |  |  |  |  |  |  |  |  |  |  |  |  |  |
| PAR  311 | A  Bone | VIII |  |  | 0/1 | 0/2 | 0/3 | 0/1 |  |  |  |  | 0/4 |  |  |  |  |  |  |  |  |  |  |  |  |  |  |  |  |
| PAR  316 | A  Bone | VIII | 0/1 | 0/1 | 0/1 | 0/2 | 0/3 | 0/1 |  |  |  |  | 0/4 |  |  |  |  |  |  |  |  |  |  |  |  |  |  |  |  |
| PAR  119 | A  Bone | VIII | 0/1 | 0/1 | 0/1 | 0/2 | 0/3 | 0/1 |  |  |  |  | 0/4 |  |  |  |  |  |  |  |  |  |  |  |  |  |  |  |  |
| PAR  120 | A  Bone | VIII |  |  | 0/1 | 0/2 | 0/3 | 0/1 |  |  |  |  | 0/4 |  |  |  |  |  |  |  |  |  |  |  |  |  |  |  |  |
| PAR  308 | A  Bone | VIII | 0/1 | 0/1 | 0/1 | 0/2 | 0/3 | 0/1 |  |  |  |  | 0/4 |  |  |  |  |  |  |  |  |  |  |  |  |  |  |  |  |
| PAR  317 | A  Bone | VIII |  |  | 0/1 | 0/2 | 0/3 | 0/1 |  |  |  |  | 0/4 |  |  |  |  |  |  |  |  |  |  |  |  |  |  |  |  |
| PAR  303 | A  Bone | VIII |  |  | 0/1 | 0/2 | 0/3 | 0/1 |  |  |  |  | 0/4 |  |  |  |  |  |  |  |  |  |  |  |  |  |  |  |  |
| PAR  315 | A  Bone | VIII | 0/1 | 0/1 | 0/1 | 0/2 | 0/3 | 0/1 |  |  |  |  | 0/4 |  |  |  |  |  |  |  |  |  |  |  |  |  |  |  |  |
| Man38 | A  Tooth | VIII | 0/1 | 0/1 | 0/1 | 0/2 | 0/2 | 0/4 |  |  |  |  | 0/3 |  |  |  |  |  |  |  |  |  |  |  |  |  |  |  |  |
|  | B Bone | II |  |  |  |  | 0/3 |  |  |  |  |  |  |  |  |  |  |  |  |  |  |  |  |  |  |  |  |  |  |
|  |  | III |  |  | 0/4 |  | 0/7 | 0/7 |  |  |  |  |  |  |  |  |  |  |  |  |  |  |  |  |  |  |  |  |  |
|  |  | VII | 0/1 | 0/1 | 0/1 |  | 0/5 | 0/1 |  |  |  |  |  |  |  |  |  |  |  |  |  |  |  |  |  |  |  |  |  |
| Man3 | A  Bone | II |  |  |  |  | 0/3 |  |  |  |  |  |  |  |  |  |  |  |  |  |  |  |  |  |  |  |  |  |  |
|  |  | III |  |  | 0/4 |  | 0/7 | 0/7 |  |  |  |  |  |  |  |  |  |  |  |  |  |  |  |  |  |  |  |  |  |
|  |  | VII | 0/1 | 0/1 |  |  | 0/5 | 0/3 |  |  |  |  |  |  |  |  |  |  |  |  |  |  |  |  |  |  |  |  |  |
| Man37 | A  Bone | I |  |  |  |  | 0/3 |  |  |  |  |  |  |  |  |  |  |  |  |  |  |  |  |  |  |  |  |  |  |
|  |  | III |  |  | 0/4 |  | 0/7 | 0/6 |  |  |  |  |  |  |  |  |  |  |  |  |  |  |  |  |  |  |  |  |  |
|  |  | IV |  |  |  |  | 0/1 |  |  |  |  |  |  |  |  |  |  |  |  |  |  |  |  |  |  |  |  |  |  |
|  |  | VI | 0/1 | 0/1 |  |  | 0/6 | 0/3 |  |  |  |  |  |  |  |  |  |  |  |  |  |  |  |  |  |  |  |  |  |
| Man9 | A  Bone | I |  |  | 0/4 |  | 0/3 |  |  |  |  |  |  |  |  |  |  |  |  |  |  |  |  |  |  |  |  |  |  |
|  |  | III |  |  |  |  | 0/7 | 0/7 |  |  |  |  |  |  |  |  |  |  |  |  |  |  |  |  |  |  |  |  |  |
|  |  | VII | 0/1 | 0/1 |  |  | 0/5 | 0/3 |  |  |  |  |  |  |  |  |  |  |  |  |  |  |  |  |  |  |  |  |  |
| Man30 | A  Bone | II |  |  |  |  | 0/3 |  |  |  |  |  |  |  |  |  |  |  |  |  |  |  |  |  |  |  |  |  |  |
|  |  | III |  |  | 0/4 |  | 0/7 | 0/7 |  |  |  |  |  |  |  |  |  |  |  |  |  |  |  |  |  |  |  |  |  |
|  |  | VI | 0/1 | 0/1 |  |  | 0/5 | 0/3 |  |  |  |  |  |  |  |  |  |  |  |  |  |  |  |  |  |  |  |  |  |
| Man2 | A  Bone | II |  |  |  |  | 0/3 |  |  |  |  |  |  |  |  |  |  |  |  |  |  |  |  |  |  |  |  |  |  |
|  |  | III |  |  | 0/4 |  | 0/7 | 0/6 |  |  |  |  |  |  |  |  |  |  |  |  |  |  |  |  |  |  |  |  |  |
|  |  | VII | 0/1 | 0/1 |  |  | 0/5 | 0/3 |  |  |  |  |  |  |  |  |  |  |  |  |  |  |  |  |  |  |  |  |  |
| Man41 | A  Bone | II |  |  |  |  | 0/3 |  |  |  |  |  |  |  |  |  |  |  |  |  |  |  |  |  |  |  |  |  |  |
|  |  | V |  |  | 0/4 |  | 0/8 | 0/7 |  |  |  |  |  |  |  |  |  |  |  |  |  |  |  |  |  |  |  |  |  |
|  |  | VII | 0/1 | 0/1 |  |  | 0/1 | 0/1 |  |  |  |  |  |  |  |  |  |  |  |  |  |  |  |  |  |  |  |  |  |
| Bös  844  (BK) | BK1 Bone | I |  |  |  |  | 0/3 |  |  |  |  |  |  |  |  |  |  |  |  |  |  |  |  |  |  |  |  |  |  |
|  |  | II |  |  |  |  |  |  |  |  |  |  |  |  |  |  |  |  |  |  |  |  |  |  |  |  |  |  |  |
|  |  | III |  |  | 0/4 |  | 0/7 | 0/7 |  |  |  |  |  |  |  |  |  |  |  |  |  |  |  |  |  |  |  |  |  |
|  |  | VII | 0/1 | 0/1 |  |  | 0/3 | 0/3 |  |  |  |  |  |  |  |  |  |  |  |  |  |  |  |  |  |  |  |  |  |
| Bös  842  (BK) | BK2 Bone | I |  |  |  |  | 0/3 |  |  |  |  |  |  |  |  |  |  |  |  |  |  |  |  |  |  |  |  |  |  |
|  |  | III |  |  | 0/4 |  |  |  |  |  |  |  |  |  |  |  |  |  |  |  |  |  |  |  |  |  |  |  |  |
|  |  | IV |  |  |  |  | 0/7 | 0/6 |  |  |  |  |  |  |  |  |  |  |  |  |  |  |  |  |  |  |  |  |  |
|  |  | VII | 0/1 | 0/1 |  |  | 0/3 | 0/3 |  |  |  |  |  |  |  |  |  |  |  |  |  |  |  |  |  |  |  |  |  |
