# Supplementary material for: Distinct Clones of Yersinia pestis Caused the Black Death
Source: PLoS Pathog. 2010 Oct 7;6(10):e1001134. doi: 10.1371/journal.ppat.1001134 (PMC2951374; doi:10.1371/journal.ppat.1001134)
Supplement: Table S4 — Summary of the results and test of the hypothesis of false negatives among the negative controls (0.03 MB DOC) [file ppat.1001134.s006.doc]

Table S4: Summary of the results and test of the hypothesis of false negatives among the negative controls. The significant tests were calculated on contingency tables obtained from the number of individuals investigated for the *pla*-gene (see Table S3). Since bone samples did not appear to contain enough bacterial DNA, for the PCR analyses only results from tooth samples were considered.

|  |  | Cases | Controls | Fisher’s exact test |
| --- | --- | --- | --- | --- |
| RDT-analysis | F1-positive | 24 | 0 | ***P*** **0.0001** |
|  | F1-negative | 23 | 28 |  |
| PCR-analysis | *pla*-positive | 10 | 0 | ***P* 0.0225** |
|  | *pla*-negative | 51 | 26 |  |
